# Pharmacology and Therapeutic Potential of Sigma_1_ Receptor Ligands

**DOI:** 10.2174/157015908787386113

**Published:** 2008-12

**Authors:** E.J Cobos, J.M Entrena, F.R Nieto, C.M Cendán, E Del Pozo

**Affiliations:** 1 Department of Pharmacology and Institute of Neuroscience, Faculty of Medicine, University of Granada, Granada, Spain; 2 Biomedical Research Center, University of Granada, Granada, Spain

**Keywords:** Sigma-1 receptors, learning and memory, depression and anxiety, schizophrenia, analgesia, pain, drugs of abuse, cocaine.

## Abstract

Sigma (σ) receptors, initially described as a subtype of opioid receptors, are now considered unique receptors. Pharmacological studies have distinguished two types of σ receptors, termed σ_1_ and σ_2_. Of these two subtypes, the σ_1_ receptor has been cloned in humans and rodents, and its amino acid sequence shows no homology with other mammalian proteins. Several psychoactive drugs show high to moderate affinity for σ_1_ receptors, including the antipsychotic haloperidol, the antidepressant drugs fluvoxamine and sertraline, and the psychostimulants cocaine and methamphetamine; in addition, the anticonvulsant drug phenytoin allosterically modulates σ_1_ receptors. Certain neurosteroids are known to interact with σ_1_ receptors, and have been proposed to be their endogenous ligands. These receptors are located in the plasma membrane and in subcellular membranes, particularly in the endoplasmic reticulum, where they play a modulatory role in intracellular Ca^2+^ signaling. Sigma_1_ receptors also play a modulatory role in the activity of some ion channels and in several neurotransmitter systems, mainly in glutamatergic neurotransmission. In accordance with their widespread modulatory role, σ_1_ receptor ligands have been proposed to be useful in several therapeutic fields such as amnesic and cognitive deficits, depression and anxiety, schizophrenia, analgesia, and against some effects of drugs of abuse (such as cocaine and methamphetamine). In this review we provide an overview of the present knowledge of σ_1_ receptors, focussing on σ_1_ ligand neuropharmacology and the role of σ_1_ receptors in behavioral animal studies, which have contributed greatly to the potential therapeutic applications of σ_1_ ligands.

## 1 HISTORICAL OVERVIEW: DISCOVERY OF SIGMA RECEPTORS AND SIGMA RECEPTOR SUBTYPES

Sigma (σ) receptors were first described as a subclass of opioid receptors [102] to account for the psychotomimetic actions of (±)-SKF-10,047 (*N*-allylnormetazocine) and other racemic benzomorphans. This early confusion was due to the complex pharmacology of this racemic compound; later studies showed that (–)-SKF-10,047 binds to µ and κ opioids, whereas the (+)-isomer lacks affinity for opioid receptors but binds to PCP (phencyclidine) binding sites with low affinity, and to a different site with high affinity, which currently retains the designation of σ [reviewed in 112 and 124] amongst others.

Two different σ sites were distinguished based on their different drug selectivity pattern and molecular mass; these two σ sites are now known as σ_1_ and σ_2_ receptors [64]. It was reported that σ_1_ binding sites display stereospecificity towards dextrorotatory isomers of benzomorphans, whereas σ_2_ binding sites display reverse selectivity, i.e., levorotatory isomers display higher affinity than dextrorotatory isomers of σ ligands [e.g. 64, 165]. The molecular weight was found to differ between the two σ receptors subtypes: the σ_1_ receptor is a 29-kDa single polypeptide first cloned in 1996 [55], whereas σ_2_ receptors have not yet been cloned and have an apparent molecular weight of 18-21.5 kDa according to photolabeling studies [65, 159]. In spite of intensive efforts in research on the σ_2_ subtype in recent years [partially reviewed in 14;  142, 156, 114], the σ_1_ subtype is much better characterized, and is the focus of this review.

Sigma_1_ receptors have been thoroughly studied in an attempt to elucidate their possible neuropharmacological applications, mainly in learning and memory processes, depression and anxiety, schizophrenia, analgesia and some effects of certain drugs of abuse. In this review we describe some aspects of the general biology of σ_1_ receptors, but focus on σ_1_ ligand neuropharmacology and the role of σ_1_ receptors in behavioral animal studies, which have contributed greatly to the understanding of the possible neuropharmacological properties of σ_1_ receptors. Non-neuropharmacological effects of σ_1_ ligands such as cardiovascular effects or their effects on cancer and immunity, and their antitussive effects, are not covered in this review. Therefore this review will not go into detail on some aspects of σ_1_ receptor knowledge, and not all references will be cited.

## 2 MOLECULAR CHARACTERISTICS, DISTRIBUTION AND PHARMACOLOGICAL PROFILE OF SIGMA_1_ RECEPTORS

### 2.1 Cloning and Structure of σ_1_ Receptors

Significant progress in our knowledge of σ receptors was made when the σ_1_ receptor was cloned. The σ_1_ receptor is a 29-kDa single polypeptide which was first cloned in guinea-pig liver [55], and later in mouse kidney, a JAR human choriocarcinoma cell line, and in the rat and mouse brain [reviewed in  54]. The protein is composed by 223 amino acids and shows the typical σ_1_ binding profile [55, 84, 180]. The amino acid sequence of the σ_1_ receptor cloned from the human cell line is highly homologous to the σ_1_ receptor cloned from the other species [181], and shows no homology with other mammalian proteins, but shares approximately 30% identity with the yeast gene that encodes the C7–C8 sterol isomerase [141], and contains an endoplasmic reticulum retention signal at the NH_2_ terminus [55, 179]. Cloning of the σ_1_ receptor has contributed greatly to research in this field, making it possible to design specific antisense oligodeoxynucleotides to study σ_1_ receptor function (as will be described below) and develop σ_1_-receptor knockout mice [91].

Several structures have been proposed for σ_1_ receptors. Initial studies proposed a single transmembrane domain structure [43, 55]. More recently, Aydar and coworkers presented evidence that the σ_1_ receptor in the plasma membrane has two transmembrane segments (when expressed in *Xenopus laevis* oocytes) with the NH_2_ and COOH termini on the cytoplasmic side of the membrane [3]. Recent studies proposed that in addition to the hydrophobic regions that constitute the putative transmembrane domains, there are two additional hydrophobic segments (one of them partially overlapping the second transmembrane domain), which were proposed to be steroid binding domain-like sites [27], and suggesting the existence of two different domains for ligand binding in the σ_1_ receptor [159], as previously proposed in earlier experiments [12]. This proposed model is illustrated in Fig. (**1**). The pharmacological characterization of these putative domains merits further study.

### 2.2 Anatomical and Subcellular Distribution of σ_1_ Receptors

#### 2.2.1 Anatomical Distribution of σ_1_ Receptors

At the anatomical level σ_1_ receptors are widely distributed in peripheral organs [e.g. 192] and different areas of the central nervous system, where they have been thoroughly studied. They are widely distributed in the brain, but concentrated in specific areas involved in memory, emotion and sensory and motor functions [reviewed in 9, 54 and 146]. In these studies high to moderate levels of σ_1_ receptors were associated with the hippocampus, especially in the dentate gyrus, hypothalamus, olfactory bulb, several cortical layers, pons, the septum, the central gray, locus ceruleus, dorsal raphe, the substantia nigra pars compacta, the red nucleus and various motor cranial nerve nuclei. The cerebellum is not particularly enriched in σ_1_ receptors, although some of its areas, such as the Purkinje cell layer, have been reported to show considerable densities of σ_1_ receptors. In addition to the brain, σ_1_ receptors are also numerous in the spinal cord, mainly in the superficial layers of the dorsal horn [2].

#### 2.2.2 Subcellular Distribution of σ_1_ Receptors

The subcellular distribution of σ_1_ receptors was firstly studied with radioligand binding in subcellular fractions, and more recently with immunochemical methods. Binding experiments with the σ_1_ radioligands [^3^H](+)-SKF-10,047, [^3^H](+)-3-PPP and [^3^H](+)-pentazocine showed that σ_1_ receptors are located in several types of mouse, rat and guinea pig brain membrane. These binding sites are more abundant in microsomal membranes, which is consistent with the endoplasmic reticulum retention signal of the cloned σ_1_ receptor [55, 179], but they are also present in nuclear, mitochondrial and synaptic membranes [17, 34, 38, 74]. Immunohistochemical studies further confirmed the existence of σ_1_ receptors in the endoplasmic reticulum not only in neurons [2], but also in many other cell types such as oligodendrocytes [160], lymphocytes [43], retinal cells [76] and certain cancer cells [62]. Detailed studies by Hayashi and Su in NG108 cells showed that σ_1_ receptors are located as highly clustered globular structures enriched in cholesterol and neutral lipids in the nuclear envelope and endoplasmic reticulum [reviewed in 62]. In neurons from the rat hypothalamus and hippocampus, electron microscopy studies showed that σ_1_ receptor immunostaining was mostly associated with neuronal perikarya, the membrane of mitochondria, some cisternae of the endoplasmic reticulum and dendrites, where it was localized in the limiting plasma membrane including the postsynaptic thickening [2].

### 2.3 Pharmacological Profile of σ_1_ Receptors: Xenobiotics and Endogenous Ligands

#### 2.3.1 Exogenous Ligands for σ_1_ Receptors

As described in the introduction, one characteristic that distinguishes σ_1_ binding sites from σ_2_ receptors is that the σ_1_ receptor displays stereospecificity towards dextrorotatory isomers of benzomorphans (such as SKF-10,047 or pentazocine) [64, 165]. An interesting aspect of σ_1_ receptor pharmacology is that these receptors can bind, with high to moderate affinity, a wide spectrum of known compounds of very different structural classes and with different therapeutic and pharmacological applications, such as neuroleptics (e.g. haloperidol, nemopramide), antidepressants (e.g. fluvoxamine, clorgyline), antitussives (carbetapentane, dextromethorphan, dimemorfan), drugs for the treatment of neurodegenerative disorders such as Parkinson’s disease (amantadine) or Alzheimer’s disease (memantine, donepezil), and drugs of abuse (cocaine, methamphetamine) (Table **1**). As for many other receptors, some allosteric modulators have been described for σ_1_ receptors, including the anticonvulsant drugs phenytoin (DPH) and ropizine (Table **1**). The modulation of σ_1_ radioligand binding by DPH has been conventionally assumed to be a characteristic difference between σ_1_ and σ_2_ binding sites [see 121 and 165 for reviews]. However, we recently reported that DPH also discriminates between different σ_1_ ligands depending on their activities on σ_1_ receptors [32, 33].

Haloperidol deserves special consideration among the σ ligands, because it is the most widely used σ_1_ antagonist in research on σ_1_ receptors, and its affinity is high enough to bind σ_1_ receptors in humans after a single oral dose [192]. In fact, haloperidol binds with similarly high affinity to dopamine D_2_ receptors and σ receptors, but its metabolites display preferential activity at σ receptors compared to dopamine D_2_ receptors [13]. Particularly interesting is the reduced metabolite of haloperidol (haloperidol metabolite II), which has high affinity for σ_1_ and σ_2_ receptors but shows much lower affinity for D_2_ receptors than the original compound [13, 108]. This compound was recently shown to be an irreversible σ_1_ ligand [34].

Some selective and high affinity σ_1_ drugs have been developed and are considered prototypical σ_1_ ligands. Examples are the σ_1_ agonists (+)-pentazocine, PRE 084, JO-1784 and SA4503, and the σ_1_ antagonists BD 1063 and NE-100. Table **1** summarizes the pharmacological activities on σ_1_ receptors, σ subtype selectivity and other known pharmacological activities of some σ ligands used in research (and also in therapeutics). Currently the number of σ ligands is increasing rapidly with the development of new compounds [35, 98, 109, 172 among others].

#### 2.3.2 Putative Endogenous σ_1_ Ligands: Neurosteroids

Although the endogenous ligands for σ_1_ receptors have not yet been defined unequivocally, currently the neurosteroids are considered the most probable endogenous σ_1_ ligands. This term, first used by Baulieu, identifies steroids that are synthesized in the central and peripheral nervous systems, and includes pregnenolone, dehydroepiandrosterone (DHEA), their sulfate esters, progesterone, and allopregnenolone [reviewed in 5]. The physiological actions of neurosteroids include genomic actions and nongenomic neuromodulatory actions, the latter of which are presumably related with σ_1_ receptors [see 146 for a detailed review]. The interaction between neurosteroids and σ_1_ receptors was first suggested in 1988 [193] from *in vitro* experiments in guinea pig brain and spleen. Of the steroids tested, progesterone was the most potent inhibitor of σ_1_-specific radioligand binding; however, whether neurosteroids are the endogenous ligands of the σ_1_ receptor remains controversial because the affinity of progesterone for σ_1_ receptors does not appear to be high enough for an endogenous ligand [178]. In addition, other steroids such as DHEAS (DHEA sulfate), pregnenolone sulfate, testosterone and deoxycorticosterone exhibited even lower affinities for σ_1_ receptors than progesterone [61]. However, some reports support that neurosteroids are the σ_1_ receptor endogenous ligands. In many experimental paradigms, progesterone behaved like other known σ_1_ antagonists, and DHEA and pregnenolone sulfate act as other known σ_1_ agonists [see 127 and 146 for an extensive review]. The exogenous administration of neurosteroids led to a dose-dependent inhibition of *in vivo* σ_1_ radioligand binding [117, 219], and modifications in endogenous levels of neurosteroids (e.g., after adrenalectomy, castration, ovariectomy or during pregnancy) affected σ_1_ responses [6, 7, 208]. The cloned σ_1_ protein presents homologies with the steroid binding site of several steroidogenic enzymes, which supports the specific interaction of σ_1_ receptors with neurosteroids [27, 127, 159]. We have therefore included them in Table **1** as putative σ_1_ endogenous ligands.

## 3 MODULATION OF CELLULAR EFFECTS BY SIGMA_1_ RECEPTORS

One of the earliest questions about the cellular effects of σ_1_ receptors concerned their possible coupling to G-proteins. This issue has been studied with different experimental approaches, and the results reported to date are as profuse as they are contradictory [reviewed in 9 and 54]. Even some selective σ_1_ agonists seemed to act through G-proteins (JO-1784), whereas others ((+)-pentazocine) did not under the same experimental conditions [143]. Now that the σ_1_ receptor has been cloned [55], it seems clear that the cloned receptor does not have the typical structure of a G-protein-coupled receptor with seven transmembrane domains; however, the existence of a metabotropic σ_1_ receptor subtype different of the cloned type cannot be ruled out yet [e.g. 104]. Although the coupling of σ_1_ receptors to G-proteins remains controversial, the modulatory role of σ_1_ receptors in the activity of some ion channels, different kinds of neurotransmission (mainly glutamatergic) and in second messenger systems, particularly the phospholipase C/protein kinase C/inositol 1,4,5-trisphosphate (PLC/PKC/InsP_3_) system, has been extensively reported.

### 3.1 Modulation of Plasmalemmal Ion Channels

#### 3.1.1 Potassium Channels

Potassium channels have been shown to constitute an important target for σ drugs. It has been shown that σ ligands inhibited K^+^ currents in several experimental preparations [97, 103, 188, 189, 220, 224]. In some of these studies,known σ_1_ agonists and antagonists produced the same effects [220, 224]. These results might reflect the participation of σ_2_ activity, since it was recently reported that σ_2_ ligands can also modulate K^+^ currents [142]. However, other recent studies showed that the selective σ_1_ agonists (+)-pentazocine and JO-1784 reduced several K^+^ currents in frog melanotropic cells [188, 189], and prevented the activation of small-conductance calcium-activated K^+^ channels (SK channels) in rat hippocampal slices [103]. These effects were reversed by known σ_1_ antagonists (NE-100 or haloperidol) [103, 188]. Regarding the molecular mechanism of the effects of σ_1_ receptors in K^+^ currents, it was proposed that σ_1_ receptors and K^+^ channels must be in close proximity for any functional interaction to occur [97, 128], and in fact the heterologous expression of σ_1_ receptors with voltage-gated K^+^ channels Kv 1.4 and 1.5, in *Xenopus* oocytes, resulted in modulation of the channel function in the absence of any σ ligand, and greater modulation in the presence of SKF-10,047 [3]. Moreover, Kv 1.4 channel not only colocalized [128] but also co-immunoprecipitated with σ_1_ receptor proteins, indicating that σ_1_ receptors are directly associated with these K^+^ channels [3]. In addition, σ_1_ photolabeling with radioiodinated probes identified high-molecular-mass protein complexes, suggesting that σ_1_ receptors may exist as oligomers or interact with protein partners either constitutively or through ligand binding [159]. The formation of these complexes might help explain the wide variety of actions produced by σ_1_ ligands in the central nervous system.

#### 3.1.2 Calcium Channels

Sigma_1_ ligands have also been reported to modulate plasmalemmal voltage-dependent calcium channels. Interaction between σ receptors and Ca^2+^ channels was suggested from studies in which the increase in intracellular Ca^2+^ concentration ([Ca^2+^]_i_) mediated by depolarization was diminished by σ ligands in neuronal cultures or forebrain synaptosomes [reviewed in 9, 54, 145]. However, in some of these experiments σ_1_ agonists and σ_1_ antagonists produced the same effects, which might be also due to the participation of σ_2_ receptors [reviewed in 145]. In addition, the selective σ_1_ agonists (+)-pentazocine and PRE 084 induced opposite effects on the increase in [Ca^2+^]_i_ induced by depolarization with KCl in NG108 cells, but both effects were reverted by σ_1_ receptor antisense oligodeoxynucleotide [59], suggesting that they were both mediated by the cloned σ_1_ receptor. It therefore seems clear that more studies are necessary to clarify the role of these receptors in plasmalemmal voltage-dependent calcium channels.

### 3.2 Neurotransmitter Systems and σ_1_ Receptors: Modulation of N-methyl-D-aspartate (NMDA) Neurotransmission

Many studies have shown that σ_1_ receptors are able to modulate several neurotransmitter systems. It has been reported that σ_1_ receptors can potentiate glutamatergic neurotransmission [partially reviewed in 9 and 146], enhance cholinergic neurotransmission [partially reviewed in 118; 71], enhance serotonergic neurotransmission [reviewed in 9], negatively modulate the GABAergic system [47, 148], diminish noradrenaline release [20], and modulate dopaminergic neurotransmission [reviewed in 124]. The direction of modulation of the dopaminergic system has been especially controversial because the contradictory results reported thus far make it difficult to reach solid conclusions. The conflicting results probably reflect the use of drugs with different degrees of selectivity for σ_1_ receptors, and different routes of administration [reviewed extensively in 124].

Among the modulatory effects on different neurotransmitter systems by σ_1_ receptors, the modulation of glutamatergic neurotransmission has been described in greater detail than others. It has been reported that σ_1_ receptors can enhance spontaneous glutamate release in the hippocampus [42, 138], potentiate glutamate release induced by brain-derived neurotrophic factor [222], potentiate the increase in [Ca^2+^]_i_ induced by glutamate in pyramidal neurons [144], and facilitate long-term potentiation in the rat hippocampus [26, 94, 103]. Of the three subtypes of glutamate-gated ion channels (NMDA, kainate and AMPA-kainate receptors), the connection between σ_1_ and NMDA receptors has been widely explored, mainly in studies of the NMDA-induced firing activity in the dorsal hippocampus. In this model σ_1_ agonists such as the selective agonists JO-1784 and (+)-pentazocine, the putative agonist DHEA, and the antidepressants clorgyline and sertraline (but not paroxetine or tranylcypromine, which showed much lower affinities for σ_1_ receptors) were able to modulate NMDA-induced firing. The effect of these ligands was reversed by known σ_1_ antagonists such as haloperidol, NE-100 or BMY-14802, and also by the putative endogenous ligand progesterone [reviewed in 9 and 146]. Interestingly, in these studies σ_1_ agonists showed a bell-shaped dose-response curve characterized by low-dose stimulation and high-dose inhibition. This type of dose-response curve indicates hormesis [18], and is well documented for σ_1_ receptor activation not only in the modulation of NMDA-induced firing, but also in many other experimental approaches, as will be described below.

Particularly interesting are the studies that related steroidal tonus under physiological conditions with the σ-mediated potentiation of glutamatergic neurotransmission in the hippocampus. This effect was strongly affected by increased levels of progesterone in pregnancy [7], and by decreased levels of this neurosteroid after ovariectomy [6]. A molecular mechanism was recently proposed by which σ_1_ receptor activation increases the NMDA receptor response. Ca^2+^ entering the cells through the NMDA receptors activates a Ca^2+^-activated K^+^ current, underlain by SK channels, which in turn shunts the NMDA receptor responses. Selective σ_1_ agonists prevented SK channel opening, and consequently increased the NMDA receptor response, emphasizing the importance of the σ_1_ receptor as a postsynaptic regulator of synaptic transmission [103]. Importantly, the modulation of several neurotransmitter systems mentioned above may be a consequence, at least partially, of the modulation of NMDA receptors. It has been reported in this connection that σ_1_ ligands can modulate dopaminergic [reviewed in 124], cholinergic [reviewed in 146], serotonergic [197] or noradrenergic systems [reviewed in 9] through NMDA receptors.

In summary, σ_1_ receptors modulate several neurotransmitter systems, and it seems that the modulation of NMDA responses by σ_1_ receptors plays a pivotal role in the modulation of neurotransmission by σ_1_ ligands.

### 3.3 Sigma_1_ Receptors as Modulators of Intracellular Messenger Systems

The modulation of metabotropic responses by σ_1_ receptors, particularly the increase in [Ca^2+^]_i_ after stimulation of InsP_3_ receptors at the endoplasmic reticulum, has been described in detail. The mechanism of modulation of the PLC/PKC/InsP_3_ system by σ_1_ receptors appears to be a complex one involving the translocation of σ_1_ receptors to the plasma membrane and the nucleus; this was proposed as a mechanism by which an intracellular receptor modulates metabotropic responses [147, 59]. Sigma_1_ receptors are localized in highly clustered globular structures associated with the endoplasmic reticulum, which contain moderate amounts of free cholesterol and neutral lipids, forming lipid droplets [62], in which σ_1_ receptor, ankyrin (specifically the ANK220 isomer) and the InsP_3_ receptors form a complex [60]. Sigma_1_ receptor activation by agonists induces the dissociation of the σ_1_ receptor-ANK220 complex from the InsP_3_ receptors [60], potentiating the calcium efflux induced by receptors that activates the PLC system (such as receptors for bradykinin and brain-derived neurotrophic factor) [59, 60, 70, 162, 222]. The enhancement of calcium efflux, which followed a bell-shaped curve [59], has been reported not only with known selective σ_1_ agonists such as PRE 084 or (+)-pentazocine [59, 60, 70], but also with other compounds such as the neurosteroids pregnenolone, pregnenolone sulfate and DHEA [59, 70], amantadine and memantine [162]. In the presence of a σ_1_ antagonist, σ_1_ receptors are dissociated from ankyrin and InsP_3_ receptors, which remain on the endoplasmic reticulum [60] where they impede the potentiation by σ_1_ agonists of bradykinin-induced Ca^2+^ efflux [59, 70, 162]. This latter effect was also prevented by the putative σ_1_ antagonist progesterone [59, 70], and by specific σ_1_ receptor antisense oligodeoxynucleotides [59]. Under basal conditions σ_1_ ligands did not affect [Ca^2+^]_i_ [59, 69], and the cells needed to be stimulated to make appropriate levels of InsP_3_ available for the modulation of [Ca^2+^]_i_ by σ_1_ receptor agonists. Additionally, the silencing of InsP_3_ receptors resulted in a decrease in σ_1_ receptor mARN levels [154], underscoring the relationship between this second messenger system and σ_1_ receptors. This proposed model of modulation by σ_1_ receptors of InsP_3_-mediated calcium efflux is illustrated in Fig. (**2**).

An additional mechanism by which σ_1_ receptors can modulate other receptors located in the plasma membrane was recently proposed. It was reported that σ_1_ receptors can affect the levels of plasma membrane lipid rafts by changing the lipid components therein [199]. This membrane reconstitution could in turn affect the function of the proteins it contains, such as neurotransmitter receptors or tropic factor receptors. In fact, σ_1_ receptors play an important role in neurite sprouting [see 62 for a more complete review].

In summary, σ_1_ receptors translocate from lipid droplets on the endoplasmic reticulum when stimulated by agonists, modulating intracellular Ca^2+^ mobilizations at the endoplasmic reticulum after activation of the PLC/PKC/InsP_3_ system, and enhancing the cellular effects of different receptors.

## 4 THERAPEUTIC POTENTIAL OF SIGMA_1_ RECEPTORS

Given the widespread distribution of σ_1_ receptors in the central nervous system and their modulatory role at cellular, biochemical and neurotransmission levels (see above), σ_1_ ligands appear to be useful in different therapeutic fields such as depression and anxiety, amnesic and cognitive deficits, psychosis, analgesia and treatment for drugs of abuse. These potential therapeutic applications are reviewed briefly below.

### 4.1 Role of σ_1_ Receptors in Learning and Memory

The central cholinergic and glutamatergic neurotransmission systems play a crucial role in learning and memory functions. Cholinergic function is disturbed in some memory pathologies such as Alzheimer’s disease and pathological ageing, in which deficits in cortical cholinergic activity have been observed [4]. In addition, NMDA receptors are involved in the induction of different forms of synaptic plasticity (such as long-term potentiation) which are thought to be the synaptic substrate for learning and memory processes [166]. As described in the section ‘Neurotransmitter systems and σ_1_ receptors: modulation of *N-*methyl-*D*-aspartate (NMDA) neurotransmission’, σ_1_ agonists facilitate long-term potentiation in the rat hippocampus. However, the administration of large doses of σ_1_ agonists or antagonists (+)-SKF-10,047, (+)-pentazocine, PRE 084, JO-1784, SA4503, DTG, BMY 14802, haloperidol, BD 1047 or NE-100, or even the downregulation of σ_1_ receptor expression by antisense oligodeoxynucleotides, failed to affect learning in control animals. This finding suggests that σ_1_ receptors are not involved in normal memory functions [see 118, 120, 121, 127, and 146 for reviews]. Bearing in mind the typical modulatory role of σ_1_ receptors, it is not surprising that they have been found to modulate memory and learning processes when a state of pharmacological or pathological imbalance is induced.

#### 4.1.1 Role of σ_1_ Receptors in Memory and Learning Impairment Induced by Drugs, Chemicals or Brain Lesions Affecting Cholinergic or Glutamatergic Neurotransmission

The learning impairment induced by the cholinergic muscarinic antagonist scopolamine, the nicotinic antagonist mecamylamine, or by cortical cholinergic dysfunction induced by ibotenic acid injection in the basal forebrain were attenuated or reversed by several σ_1_ agonists, including the selective σ_1_ agonists (+)-pentazocine, JO-1874 and SA4503 [reviewed in 118, 121 and 146]. In addition, the memory impairments induced by the serotonin (5-HT) depleter *p*-chloroamphetamine (PCA), which also involves cholinergic dysfunction [115], were attenuated in a bell-shaped manner by the administration of (±)-pentazocine, (+)-3-PPP, DTG, and (+)-SKF-10,047 [115, 116]. The effects of σ_1_ agonists in scopolamine-induced amnesia were reversed by known σ_1_ antagonists including haloperidol and NE-100, and by the downregulation of σ_1_ receptor expression by specific antisense oligodeoxynucleotides (reviewed in 118, 121 and 146). Interestingly, the putative σ_1_ agonists pregnenolone sulfate and DHEAS were also effective in scopolamine-induced amnesia model, and their effects were reversed by NE-100 and progesterone [120, 121 and 146].

As noted above, NMDA receptors also play an important role in learning and memory processes. The σ ligands (+)-SKF-10,047, (+)-pentazocine, JO-1784, DTG, PRE 084 and SA4503, and also the putative endogenous σ_1_ agonists DHEAS and pregnenolone sulfate attenuated the learning deficits induced by dizocilpine (MK-801), a noncompetitive NMDA receptor antagonist, in rats and mice presented with different mnesic tasks. The anti-amnesic effect of σ_1_ agonists was reverted by several known σ_1_ antagonists such as haloperidol, BMY 14802, NE-100 and BD 1047, by the putative endogenous σ_1_ antagonists progesterone [partially reviewed in 120, 121 and 146; 127], and by the administration of antisense oligodeoxynucleotides against σ_1_ receptors [122, 123, 126]. Cholinesterase inhibitors such as rivastigmine, tacrine and donepezil also attenuated dizocilpine-induced learning impairments [126]; however, only the effect of donepezil (which is also a potent σ_1_ ligand, see Table **1**) was blocked by BD 1047 or antisense treatment [126].

Repeated exposure to carbon monoxide (CO) gas induced long-lasting but delayed amnesia which was measurable about one week after exposure. Like models of ischemia, this model involves the neurotoxicity of excitatory amino acids, and the hippocampal cholinergic system appears markedly affected by hypoxic toxicity [reviewed in 118]. Sigma_1_ ligands have been shown to have neuroprotective properties in models of ischemia [partially reviewed in 118, 16, 81]. Consistent with this neuroprotective action is the observation that the σ ligands (+)-SKF-10,047, PRE 084, JO-1784 and DTG reversed CO-induced amnesia, and their effects were prevented by NE-100, BMY 14802 and BD 1047 [partially reviewed in 120, 135]. Donepezil and some other cholinesterase inhibitors have also been tested in this behavioral model of amnesia, and it was found that all drugs showed anti-amnesic properties, but the pre-administration of BD 1047 blocked only the effect of donepezil [135]. Interestingly, in this model of amnesia the σ_1_ antagonists BD 1008 and haloperidol also showed anti-amnesic effects that were not reversed by NE-100, so it was suggested that these drugs might produce their effects through their σ_2_ agonistic activity [120]. The role of σ_1_ receptors in these experimental models is summarized in Table **2**.

#### 4.1.2 Role of σ_1_ Receptors in Cognitive Impairments in Ageing: Alzheimer Disease

In models related with the memory deficits of ageing, σ_1_ agonists were also effective in attenuating the learning deficits in aged mice, aged rats and in senescence-accelerated mice, [reviewed in 118, 146]. Moreover, in the model of Alzheimer’s disease-type amnesia induced by β_25-35_-amyloid related peptide, which involves both cholinergic and glutamatergic neurotransmission through NMDA receptors [121], the σ_1_ receptor agonists (+)-pentazocine, PRE 084, SA4503, (+)-SKF-10,047, the antitussive drug dimemorfan and the putative σ_1_ agonists DHEAS and pregnenolone sulfate attenuated amnesia in a bell-shaped manner. The effects of σ_1_ agonists were reverted by haloperidol, BD 1047 and the putative σ_1_ antagonist progesterone [119, 137, 217]. Donepezil and other cholinesterase inhibitors were also tested in this behavioral model, but only the effects of donepezil were partially reversed by BD 1047, suggesting that the anti-amnesic effects of this drug involve both its cholinergic and σ_1_ agonistic properties [137]. These findings are consistent with the neuroprotective action of the σ_1_ agonist PRE 084, which attenuated cell death in cultured cortical neurons co-incubated with β_25-35_-amyloid related peptide, and this effect was reversed by the selective σ_1_ antagonist NE-100 [99]. The effects of σ_1_ ligands in ageing-related cognitive impairment are summarized in Table **2**.

#### 4.1.3 Other Ameliorative Effects of σ_1_ Agonists on Learning and Memory

Stress during pregnancy directly affects the neurophysiological development of the fetus with deleterious consequences observable throughout the individual’s lifetime [89], and can result in impairments in learning and memory processes [134]. The σ_1_ agonist JO-1784 reversed the learning deficits induced by prenatal stress, in a BD 1063-sensitive manner [134]. In addition, it is known that repeated cocaine treatment *in utero* can induce learning and memory impairment in the offspring. It was recently found that this process can be reverted by the σ_1_ agonist JO-1784 or DHEA, in a BD 1063-sensitive manner [133]. On the other hand, cocaine administered at very low doses (much lower doses than those which induce learning and memory impairments) can enhance memory storage in mice [72]. The ameliorating effects of cocaine on memory can be enhanced by the σ_1_ agonist JO-1784 and also by the putative σ_1_ agonist DHEA, and masked by the σ_1_ antagonist BD 1047 and also by the putative σ_1_ antagonist progesterone [171]. The hyperlocomotion, toxic effects, and reward properties induced by this psychostimulant are observed at much higher doses, and the effects of σ_1_ ligands on these effects will be described later in section 4.5.1 on cocaine and σ_1_ receptors. These effects of σ_1_ agonists on cognitive impairment due to alterations during pregnancy, as well as their role in the ameliorative effects of low doses of cocaine, are summarized in Table **2**.

In summary, σ_1_ agonists appear to be promising pharmacological tools against memory and learning disorders resulting from pharmacological or pathological alterations (see Table **2**). Among the memory and learning disorders, Alzheimer’s disease (the most common form of late-life dementia) is characterized by a cognitive decline, and effective treatment remains elusive. Sigma_1_ agonists could thus provide an alternative treatment against the cognitive deficits of this disease.

### 4.2 Role of σ_1_ Receptors in Depression and Anxiety

Several neurotransmitter systems are important in the pathophysiology of depression and anxiety. Depression likely involves dysfunction in brain areas that are modulated by monoaminergic systems such as the frontal cortex and the hippocampus [reviewed in 39]. Given that σ_1_ ligands play a modulatory role in several neurotransmitter systems, and that they can bind several known antidepressants (Table **1**), they have been studied as possible pharmacological tools against these mood disorders.

#### 4.2.1 Depression and σ_1_ Receptors

The effects of σ_1_ ligands were tested in behavioral studies used to predict the antidepressant activity of drugs. The selective σ_1_ agonists SA4503 and (+)-pentazocine decreased immobility time in the tail suspension test, and this effect was antagonized by NE-100 [207]. Many σ_1_ agonists have been tested in the forced swimming test, for example SA4503, (+)-pentazocine, JO-1784, DHEAS, pregnenolone sulfate, donepezil, and some novel σ selective compounds such as UMB23, among others. The decrease in immobility in the forced swimming test induced by the σ_1_ agonists was blocked by known σ_1_ antagonists [partially reviewed in 121; 126, 185, 208, 210, 218]. Interestingly, the extracts of the flowering plant *Hypericum perforatum* (St. John’s wort), which are used as antidepressants, appear to exert their therapeutic actions through σ_1_ receptors [reviewed in 132]. Additional experiments have related endogenous neurosteroidal levels with σ_1_ receptor function. In adrenalectomized and castrated mice, the effect of JO-1784 and PRE 084 in the forced swimming test was enhanced compared to control animals, and these effects were blocked by the selective σ_1_ antagonist BD 1047 [208]. In addition, the antidepressant efficacy of the selective agonist JO-1784 was enhanced in 12-month-old senescence-accelerated (SAM) mice, which showed decreased levels of progesterone [163]. Moreover, in animals acutely treated with β_25-35_-amyloid related peptide, which does not modify their immobility time, the effects of the selective σ_1_ agonists JO-1784 and PRE 084 were facilitated, presumably because of a decrease in progesterone levels in the hippocampus [209].

An important consideration is that σ_1_ agonists were able to potentiate the firing of serotonergic neurons of the dorsal raphe nucleus, as early as after 2 days of treatment, whereas SSRI- and monoamine oxidase inhibitor-induced changes took several weeks to emerge. The rapid effect of σ_1_ agonists has been proposed to predict a more rapid onset of antidepressant efficacy compared to existing medications [8]. Because of the typically modulatory role of σ_1_ receptors, OPC-14523, a compound with high affinity for σ_1_ receptors, 5-HT_1A_ receptors, and serotonin transporter (SERT) (Table **1**) was developed, and was found to produce a marked antidepressant-like effect in the forced swimming test after a single oral administration. This effect was reversed by both σ_1_ and 5-HT_1A_ antagonists [203]. Moreover, and also in keeping with the modulatory role of σ_1_ receptors, the combined administration of the selective σ_1_ receptor agonist (+)-pentazocine and venlafaxine [41], or the co-administration of pramipexole and sertraline [167], at subeffective doses, showed a synergistic antidepressant-like effect, as did the co-administration of SA4503 and memantine or amantadine [186]. Importantly, the antidepressant-like effects of these drugs were reversed by known selective σ_1_ antagonists, and also by progesterone [41, 167, 186].

It is known that NMDA receptor subunit 1 is decreased in the prefrontal cortex or hippocampus of depressive patients [92, 155]. In the olfactory bulbectomized rat model of depression, animals show a decrease in NMDA receptor subunit 1 in these areas, and exhibit behavioral deficits which resemble the psychomotor agitation, loss of interest, and cognitive dysfunction of depression. Repeated treatment with SA4503 ameliorated the behavioral deficits, and also reversed the decrease in the protein expression of NMDA receptor subunit 1. These effects of SA4503 were blocked by the co-administration of NE-100 and by acute treatment with the NMDA receptor antagonist dizocilpine [216]. These findings document the strong relationship between depression, NMDA receptors and σ_1_ receptors.

In addition to the modulatory role of σ_1_ receptors in NMDA- and 5-HT-mediated responses related with depression, a complementary mechanism of action of σ_1_ ligands in this disorder has been reported to involve their effects in neuroplasticity processes. The mechanism of action of some antidepressants may involve neurotrophic actions [150], and it was reported that treatment with (+)-pentazocine or the antidepressants imipramine and fluvoxamine (which exhibit affinity for σ_1_ receptors, see Table **1**) enhanced growth factor-induced neurite sprouting in PC12 cells, and also upregulated σ_1_ receptors [198]. The enhancement of growth factor-induced neurite sprouting by these drugs was mimicked by the overexpression of σ_1_ receptors [199].

In humans σ_1_ receptors can bind fluvoxamine at therapeutic doses [73], suggesting that this receptor might mediate some of the effects of this antidepressant; it has also been reported that JO-1784, at doses of 20 mg/day, exhibited a stronger antidepressant effect than the known antidepressant fluoxetine at the same dose in clinical trials. However, at 100 mg/day JO-1784 was no different from the placebo [9], which is in keeping with the bell-shaped dose-response curves induced by σ_1_ agonists in several behavioral, biochemical, and electrophysiological paradigms.

In summary, σ_1_ agonists showed good antidepressant effects in several behavioral models, probably because of their enhancement of serotonergic and glutamatergic neuronal functions as well as their neurotrophic actions (see Table **3**). Due to the typically modulatory role of σ_1_ receptors, the design of drugs with mixed affinity for σ_1_ and other receptors related with depression, and the combined treatment of σ_1_ agonists with known antidepressant drugs, may offer good prospects in terms of efficacy.

#### 4.2.2 Anxiety and σ_1_ Receptors

Evidence of anxiolytic activity of σ_1_ ligands was reported in the conditioned fear stress model, in which (+)-SKF-10,047, JO-1784, the neurosteroids pregnenolone sulfate and DHEAS, and also the antitussive dextromethorphan attenuated the motor suppression induced by previous electric footshock [79, 80, 153, 211], in a bell-shaped manner [211]. In addition, the effects of σ_1_ agonists on motor suppression were reversed by the known σ_1_ antagonist NE-100 and progesterone [153, 211]. In contrast, (+)-pentazocine lacked any effect in this model [79, 80]. Interestingly, the concentration of σ_1_ active steroids was altered in the plasma and brain of stressed mice, and it was therefore hypothesized that endogenous levels of neurosteroids might be involved in the expression of conditioned fear stress responses *via* σ_1_ receptors [153, 211]. In agreement with these results, animals treated chronically with β_1-40_-amyloid related peptide, in which progesterone levels in the hippocampus and cortex were decreased, exhibited facilitation of the effect of the σ_1_ agonists JO-1784, (+)-SKF-10,047 and DHEAS [211].

The effects of σ_1_ ligands have also been assayed in other behavioral tests such as sexual dysfunction induced by stress, marble-burying behavior and colonic motor disturbances induced by fear. It was reported recently that DHEA attenuated stress-induced sexual dysfunction in rats in a NE-100 dependent manner [140]. In the marble-burying behavior test, considered a potential model of obsessive–compulsive disorder on the basis of behavioral similarity, the effect of fluvoxamine was antagonized by BD 1063 and BD 1047, but not by the σ_2_ antagonist SM-21, suggesting again that the interaction of fluvoxamine with σ_1_ receptors contributes to its antidepressant effects. In addition, the σ_1_ agonists (+)-SKF-10,047 and PRE 084 slightly inhibited marble-burying behavior [44]. Gue and coworkers [52] showed that JO-1784 suppressed stress-induced colonic motor disturbances induced by fear stress in rats, in a model that mimicked the gastrointestinal tract disorders frequently present in anxiety, and this effect was reversed by BMY 14802 [52]. Subsequently, JO-1784 showed good results in clinical trials in a phase-1-model of functional diarrhea [213]. The results described above (summarized in Table **4**) suggest that σ_1_ receptors play an important role in the modulation of anxiety.

### 4.3 Schizophrenia and σ_1_ Receptors

The dopamine hypothesis of schizophrenia, which involves enhanced mesolimbic dopamine function, remains the dominant hypothesis for the pathophysiology of this disorder, particularly regarding the appearance of positive symptoms [40]. In addition, it is important to consider the glutamatergic system. In fact, the blockade of NMDA receptors by PCP induces schizophrenia-like psychosis in humans [24, 212]. Because several antipsychotics possess high to moderate affinities for σ_1_ receptors (Table **1**), researchers were inspired to test σ_1_ receptor ligands in several animal models of schizophrenia.

#### 4.3.1 Role of σ_1_ Receptors in Behavioral Models of Schizophrenia in which Dopaminergic Function is Prominently Enhanced

In behavioral animal models in which the dopaminergic function is affected, such as apomorphine-induced climbing, amphetamine-induced locomotor activity, and behavioral sensitization by the repeated administration of psychostimulants, promising results have been reported using σ_1_ antagonists (summarized in Table **5**). The nonselective σ_1_ antagonist BMY 14802, panamesine, E-5842 and MS-377 inhibit apomorphine-induced climbing [53, 187, 195, 201]. In addition, DTG, SR 31742A, panamesine, rimcazole and E-5842 inhibit amphetamine-induced locomotor activity [53, 164, 176, 187]. However, rimcazole and BD 1047 had little effect on apomorphine-induced climbing, and in addition, this latter compound had little effect on acute amphetamine-induced hyperlocomotion [187]. In models of behavioral sensitization with the repeated administration of psychostimulants—a pharmacological model of schizophrenia [21]— σ_1_ antagonism inhibited sensitization to methamphetamine [1, 196, 205] and cocaine [206, 221]. It was therefore suggested that σ_1_ antagonists may be suitable for maintenance therapy in persons with stable schizophrenia rather than for the treatment of acute psychotic features.

#### 4.3.2 Role of σ_1_ Receptors in Behavioral Models of Schizophrenia in which Glutamatergic Function is Prominently Disturbed

As said before, in addition to dopaminergic dysfunction, alterations in glutamatergic neurotransmission are also involved in schizophrenia. Sigma_1_ ligands modified animal behavior in some glutamatergic models of this disease (summarized in Table **5**). PCP-induced head weaving, which is insensitive to selective D_2_ antagonists, was attenuated by NE-100, haloperidol, BMY 14802, Dup 734 and MS-377 [63, 195]. Recent reports also showed that BD 1047, rimcazole and panamesine attenuated PCP-induced head twitching [187]. In addition, selective σ_1_ receptor agonists such as (+)-pentazocine, and also 3-(+)-PPP and (+)-SKF-10,047, enhanced the psychotomimetic effect (hyperlocomotion) of dizocilpine in monoamine-depleted mice, and this enhancement was blocked by NE-100 [157], suggesting that σ_1_ receptor blockade may be effective for negative symptoms of schizophrenia, which are hypothesized to be mediated, at least in part, by glutamatergic neurotransmission. Among the negative symptoms of schizophrenia, cognitive deficits are core features of the illness and predict vocational and social disabilities for patients [90]. It has been extensively reported that σ_1_ agonists play an important role in memory processes (as described in the section 4.1., ‘Role of σ_1_ receptors in learning and memory’). In fact, SA4503, DHEAS, and fluvoxamine (a SSRI with high affinity for σ_1_ receptors), but not paroxetine (an SSRI without affinity for σ_1_ receptors) improved the PCP-induced cognitive deficits in the novel object recognition test, and these effects were antagonized by the co-administration of NE-100 [58]. In addition, the antipsychotic (and also σ_1_ antagonist) drug haloperidol was ineffective in this behavioral model [57]. These results suggest that σ_1_ agonists are potentially useful for the cognitive deficits of schizophrenia.

#### 4.3.3 Sigma_1_ Receptors and Extrapyramidal Side Effects

The extrapyramidal effects of neuroleptics are considered one of the most problematic side effects of these drugs. It has been suggested that σ receptors mediate the undesirable motor side effects of antipsychotic drugs [reviewed in 54 and 214], an effect classically attributed to the σ_2_ subtype [e.g.,^ 215^]. Although it was found that the affinities of several neuroleptics for σ receptors (both σ_1_ and σ_2_) correlated well with their risk of producing acute dystonic reactions [108], it is known that the blockade of σ_1_ receptors with other more selective antagonists such as NE-100 [158], MS-377 [195], E-5842 [53] or BMY 14802 [50] (at effective doses for the test used) does not induce motor side effect. These findings suggest that the blockade of σ_1_ receptors is not enough in itself to induce extrapyramidal side effects, so additional mechanisms are probably be involved.

#### 4.3.4 Clinical Trials with σ_1_ Ligands in Schizophrenia

Some clinical trials have been done with rimcazole, BMY 14802, eliprodil (SL-82.0715) and panamesine. The trials with rimcazole and BMY 14802 yielded inconclusive results [reviewed in 61]; however, eliprodil reduced scores for negative but not positive symptoms, whereas panamesine reduced both positive and negative symptoms. However, a metabolite of panamesine has potent antidopaminergic properties which might explain its effect against the positive symptoms, so further research is needed to determine whether these effects are wholly or partly mediated by σ_1_ receptors [reviewed in 61].

In summary, due to the complex pathogenesis of schizophrenia and the differential effects of σ_1_ antagonists (which improve the behavior of animals in models based on the motor effects of dopaminergic stimulants or NMDA antagonists) and σ_1_ agonists (which improve the cognitive deficits induced by PCP) (summarized in Table **5**), treatment based exclusively on σ_1_ ligands would probably be complex.

### 4.4 Sigma_1_ Receptors and Analgesia

Sigma_1_ receptors are distributed in the central nervous system in areas of great importance in pain control, such as the superficial layers of the spinal cord dorsal horn, the periaqueductal gray matter, the locus ceruleus and rostroventral medulla [2, 88]. As will be described below, they may be involved in the modulation of opioid analgesia, and may also play an important role in nociception in the absence of opioid drugs.

#### 4.4.1 Modulation of Opioid Analgesia by σ_1_ Receptors

Chien and Pasternak were the first to report the involvement of σ_1_ receptors in analgesia [28]: they clearly demonstrated that σ_1_ receptors play an important role in the modulation of opioid analgesia in the tail-flick test. The systemic administration of σ_1_ agonists, including the selective σ_1_ agonist (+)-pentazocine, antagonized the antinociception induced by morphine in the tail-flick test [28-30, 130]. Further experiments with other opioids confirmed the role of σ_1_ receptors in opioid analgesia. (+)-Pentazocine also diminished δ-, κ_1_, and κ_3_ opioid antinociception [29, 130, 173]. In addition, σ_1_ antagonists such as haloperidol and (+)-MR 200 not only reversed the effects of agonists, but also increased opioid-induced antinociception, indicating the presence of a tonically active anti-opioid σ_1_ system [28-30, 100, 173].

The anatomical location of the modulation of opioid analgesia by σ_1_ receptors has been determined with different routes of administration of opioids, σ_1_ receptor ligands and antisense oligodeoxynucleotides. The intrathecal (i.t.) administration of (+)-pentazocine did not reverse the spinal (i.t.) analgesic effect of morphine in the tail-flick test, suggesting that the modulation of opioid analgesia by σ_1_ receptors in this test does not occur at the spinal level [130]. Interestingly, the supraspinal (intracerebroventricular, i.c.v.) administration of (+)-pentazocine decreased the analgesic effect of agonists for the κ and μ opioid receptors nalorphine and nalbuphine [130]; in addition, the analgesia induced by the supraspinal (i.c.v.) administration of the selective µ-opioid agonist DAMGO was enhanced by the σ_1_ antagonist (+)-MR 200 administered subcutaneously [100]. Further experiments based on the selective blockade of σ_1_ receptor synthesis by the i.c.v. administration of specific antisense oligodeoxynucleotides confirmed the supraspinal location of the modulation of opioid analgesia [87, 130, 161]. Finally, a more detailed approach was tested recently by Mei and Pasternak [131], who used microinjections of morphine in conjunction with (+)-pentazocine, haloperidol, or both in three brainstem nuclei: the periaqueductal gray, rostroventral medulla and locus ceruleus. The activity of σ_1_ receptors was found to differ depending on the area. Whereas both the locus ceruleus and rostroventral medulla were sensitive to (+)-pentazocine, the periaqueductal gray was not. The rostroventral medulla was particularly interesting, because it was the only region with evidence for tonic σ_1_ activity (enhanced by haloperidol), and it was also able to modulate the analgesia from morphine administered to the periaqueductal grey.

In contradistinction to results in the tail-flick test, it was found that the systemic administration of (+)-SKF-10,047 or NE-100 was unable to modulate κ_1_ opioid analgesia in the acetic acid-induced writhing test [66]. Although the doses used in this study might have been too low to prevent the participation of σ_1_ receptors in the modulation of κ_1_ opioid analgesia in the acetic acid-induced writhing, i.c.v. treatment with σ_1_ antisense oligodeoxynucleotides also failed to affect this response [67], suggesting that the supraspinal inhibition of σ_1_ receptors does not affect κ opioid analgesia in this behavioral test. This findings may indicate that the supraspinal σ system modulates only some opioid analgesic effects, probably depending on the type of pain evaluated (i.e., depending on the behavioral model used). Further research with different models is needed to characterize the role of the supraspinal σ system in opioid analgesia. The role of σ_1_ receptors on opioid analgesia in behavioral experimental models is summarized in Table **6**.

In addition, some recent reports showed that haloperidol and chlorpromazine, two neuroleptics that bind to σ sites (Table **1**), inhibit the antianalgesia induced by nalbuphine in men [48]. Although the authors did not attribute this inhibition to σ receptors, this possibility cannot be fully ruled out, and would suggest that interaction between the σ and opioid systems is important in clinical terms. However, this issue also needs to be addressed in further clinical studies.

#### 4.4.2 Analgesic Effect of σ_1_ Receptor Ligands

The role of σ_1_ ligands in the absence of opioid drug has also been investigated. Several σ_1_ ligands or antisense treatments have been proved to be inactive in the tail-flick test [22, 28-30, 100, 130, 161], as well as in the acetic acid-induced writhing test [66, 67] (although higher doses of σ_1_ ligands should be tested to ensure their lack of involvement in acetic acid-induced writhing). However, other reports showed that σ_1_ receptors are able to modulate nociception in other behavioral tests in the absence of an opioid drug. Ueda and coworkers [204] showed that the σ_1_ agonists (+)-pentazocine and SA4503, (+)-3-PPP, and also the putative σ_1_ agonists DHEAS and pregnenolone sulfate (administered intraplantarly) can induce nociception even when used alone in the nociceptive flexor response test. The effect of the σ_1_ agonists was reverted by the known σ_1_ antagonists NE-100, BD 1047 or the putative σ_1_ endogenous antagonist progesterone [204]. Other studies in our laboratory with the formalin test showed that formalin-induced nociception was attenuated not only by the systemic administration of haloperidol, haloperidol metabolite II and haloperidol metabolite I (with an order of potency which correlated with their affinity for σ_1_ receptors) [22], but also in σ_1_ receptor knockout mice [23]. Recent experiments with the same behavioral test found that in contradistinction to the supraspinal action of σ_1_ antagonists on the modulation of opioid analgesia, the i.t. administration of the σ_1_ receptor antagonists BD 1047 and BMY 14802 dose-dependently reduced formalin-induced pain behaviors in the second phase but not in the first phase of the formalin test [86]. This underscored the importance of spinal σ_1_ receptors in the second phase of formalin-induced pain. These results were consistent with previous findings which showed that haloperidol, haloperidol metabolite I and haloperidol metabolite II were more effective in the second than in the first phase of formalin-induced pain [22]. In agreement with these behavioral studies, it was also reported that antagonism of spinal σ_1_ receptors suppressed phosphorylation of the NR1 subunit of spinal NMDA receptors [86], which are important for maintaining spinal sensitization associated with the second phase of the formalin test [190]. From these results it was proposed that σ_1_ receptors may be important in models in which spinal sensitization occurs (without ruling out other analgesic effects in other models), and in fact, the putative σ_1_ agonist DHEA induced mechanical allodynia and thermal hyperalgesia when administered i.t., and the effects were reversed by BD 1047 [85]. This hypothesis deserves further investigation in other models of pain, especially in models of tonic pain in which central sensitization occurs. The results obtained in the behavioral models described above (summarized in Table **6**) suggest that σ_1_ receptors play an important role in nociception in the absence of opioid drugs.

In summary, σ_1_ receptors are not only able to modulate opioid antinociception, at least in the tail-flick test, but may also play an active role in nociception in the absence of opioid drugs in some behavioral models (see Table **6**). 

### 4.5 Sigma_1_ Receptors and Drugs of Abuse

As shown before (Table **1**), σ_1_ receptors can bind several drugs of abuse. It is therefore not surprising that σ_1_ ligands can modulate some of the effects of these drugs. Among the drugs of abuse studied to date, the involvement of σ_1_ receptors in the actions of cocaine has been extensively investigated, but σ_1_ receptors also appear to underlie the effects of other substances such as methamphetamine, MDMA (3,4-methylenedioxymethamphetamine) and ethanol, as will be described below.

#### 4.5.1 Cocaine and σ_1_ Receptors

Cocaine is generally thought to act as a dopamine reuptake inhibitor to produce its reinforcing effects, although other mechanisms might also be important [105]. Cocaine binds preferentially to σ_1_ receptors rather than to σ_2_ [111], and the affinity of cocaine for σ_1_ receptors is in the micromolar range (Table **1**), as is its affinity for its main pharmacological target, the dopamine transporter (DAT) [175]. Cocaine levels in the post-mortem brain of addicts were estimated to be between 0.1 and 4 µM [78], which is close to the *K*_i_ value of cocaine for σ_1_ receptors. In recent years several excellent and promising studies have been performed with σ_1_ ligands against the effects of cocaine, as described below.

##### 4.5.1.1 Modulation by σ_1_ Receptors of the Acute Effects of Cocaine 

The ability of compounds to attenuate the acute locomotor effects of cocaine is often used as an initial screening tool to identify agents able to block the psychostimulant activity of this drug of abuse. Convulsions and lethality, on the other hand, represent a measure of cocaine toxicity, and can result from exposure to acute large doses. Many σ_1_ antagonists have been reported to significantly prevent the acute locomotor stimulatory effects, convulsions or lethality induced by cocaine in rodents, including haloperidol, BD 1008 (and some of its analogs such as the selective σ_1_ antagonists BD 1047 and BD 1063), BMY 14802, panamesine and rimcazole (and some of its analogs), among others [partially reviewed in 112 and 124; 37, 95, 113]. Furthermore, the administration of antisense oligodeoxynucleotides that knock down brain σ_1_ receptors mimicked the effects of pharmacological σ_1_ antagonism on the locomotor stimulatory effects or convulsions induced by cocaine [109, 111]. Particularly interesting are the studies in which post-treatment of mice with the novel σ receptor antagonists LR132 and YZ-011, after cocaine administration, also attenuated cocaine-induced lethality after an overdose. However, BD 1063 was unable to prevent death under these conditions, and the authors hypothesized that this result was due to differences in pharmacokinetics [109, 111]. The ability of σ receptor antagonists to prevent death after an overdose of cocaine in animals suggests a clinical application potentially worth further study. In contradistinction to the positive effects of σ_1_ antagonists, the administration of DTG, the novel σ_1_ agonists BD1031 and BD1052, or the selective σ_1_ agonist SA4503 exacerbated locomotor stimulatory actions and the toxic effects (measured as convulsions and lethality rate) of the acute administration of cocaine [109, 111, 129, 184]. The results obtained in these behavioral models (summarized in Table **7**) suggest that σ_1_ receptors play an important role in the acute effects of cocaine. In addition to σ_1_ receptors, it has been proposed that the σ_2_ subtype might also be a good pharmacological target against cocaine-induced actions [partially reviewed by 113; 114, 156].

##### 4.5.1.2 Modulation by σ_1_ Receptors of the Effects of Repeated Cocaine Administration

Several σ_1_ antagonists have also been tested in behavioral models that used repeated doses of this drug of abuse. The σ_1_ antagonists rimcazole and some of its analogs, and other putative σ antagonists did not alter or only slightly altered the discriminative stimulus of cocaine [83, 95, 221], indicating that the interaction between cocaine and σ receptor ligands might be more complex than an exclusively competitive antagonism. Other studies that involved the repeated administration of cocaine found that σ receptor antagonists significantly prevented the development of cocaine-induced locomotor sensitization [206, 221], which is considered a measurable index of nervous system plasticity resulting from repeated exposure to cocaine [112]. The effects of σ_1_ antagonism on the rewarding properties of this drug of abuse have been explored with promising results. In the conditioned place preference test, the selective σ_1_ receptor antagonists BD 1047 and NE-100 attenuated the acquisition [168, 169] and also the expression of cocaine-induced conditioned place preference [169]. In addition, treatment with σ_1_ antisense oligodeoxynucleotide was effective against the acquisition of conditioned place preference, indicating the specificity of these effects [168]. However, in cocaine self-administration experiments, Martin-Fardon and coworkers found that BD 1047 was inactive against the acute reinforcing effects of cocaine, supposedly because both the reinforcing quality and relevant neuroadaptive changes are likely to differ in rats subjected to involuntary administration (as in conditioned place preference) vs. self-administration of cocaine [101]. After extinction, cocaine addictive behavior can be reactivated by a discriminative stimulus associated with cocaine administration, or by a priming injection of cocaine (in self-administration or conditioned place preference experiments, respectively). These processes were both blocked by BD 1047 [101, 170], and the latter one was also blocked by σ_1_ antisense oligodeoxynucleotides [170]. Interestingly, the σ_1_ agonists PRE 084 and JO-1784 were unable to induce conditioned place preference [169], but the administration of the latter σ_1_ agonist, or even DHEA, was enough to reactivate conditioned place preference after extinction, in a BD 1047-sensitive manner [170]. The results in these behavioral models (summarized in Table **7**) suggest that σ_1_ receptors play an important role in neuronal plasticity after repeated cocaine administration, and that σ_1_ antagonists could be useful to prevent craving and relapse of cocaine addiction.

It has been reported that σ_1_ receptor density changes after repeated treatment with cocaine [96, 169, 170, 183, 223]. Particularly interesting is the σ_1_ receptor upregulation in the caudate putamen (an important area in the drug reward mechanism), which was not produced in dopamine D_1_ receptor knockout mice [223]. Consistent with this finding was that cocaine treatment in the neuroblastoma cell line B- 104 (lacking in dopamine transporter or receptors), was also unable to induce σ_1_ receptor upregulation [36], suggesting a close relationship between dopamine and σ_1_ receptors. In fact, it has been proposed that both D_1_ receptors and σ_1_ receptors are involved in cocaine-induced life-long alterations in neurons [194].

##### 4.5.1.3 Effects of σ_1_ Ligands on Cocaine-Induced Immune System Depression

Different experiments have been designed to investigate the effects of cocaine other than its acute toxicity or rewarding properties, specifically, modulation of the immune system by cocaine. It was recently reported that cocaine can enhance alveolar cell carcinoma growth in mice, and that this effect was mimicked by PRE 084 and reversed by BD 1047. Increased tumor growth induced by cocaine or PRE 084 was accompanied by an increase in IL-10 and a decrease in IFN-γ production [46]. In addition, the selective σ_1_ antagonist BD 1047 blocked enhancement of the replication of HIV-1 in mice with severe combined immunodeficiency implanted with HIV-1-infected human peripheral blood mononuclear cells [174], and also in human microglial cell cultures [49]. These reports suggest that σ_1_ receptors are involved in the cocaine-induced depression of the immune system.

In summary, σ_1_ antagonists appear to be potentially useful not only against acute cocaine toxicity or addiction, but also against the noxious modulation of the immune system in cocaine consumers. In addition, σ_1_ agonists, as described in section 4.1. ‘Role of σ_1_ receptors in learning and memory,’ may be useful against some behavioral alterations induced by repeated cocaine exposure *in utero*. It therefore seems clear that cocaine produces its behavioral and biochemical effects, at least in part, through its interaction with σ_1_ receptors, and that σ_1_ ligands should be considered for the development of potential therapies to treat different aspects of cocaine abuse.

#### 4.5.2 Other Drugs of Abuse and σ_1_ Receptors

Methamphetamine, like cocaine, also binds to σ_1_ receptors in the micromolar range (Table **1**), and with a 20-fold higher affinity than for σ_2_ receptors [151], so it is not unexpected that σ_1_ ligands modulate some effects of this psychostimulant. Early studies found that the σ_1_ antagonists NE 100, BMY 10802 and MS-377 modulated the acute motor effects of methamphetamine only weakly, if at all [158, 196, 205]. However, more recent studies showed that the selective σ_1_ antagonists BD 1063 and BD 1047, as well as σ_1_ antisense oligodeoxynucleotide, inhibited methamphetamine-induced locomotor activity [151]. It was also recently reported that like cocaine or methamphetamine, the compound MDMA (‘ecstasy’), which is structurally related to methamphetamine, showed preferential affinity for σ_1_ receptors rather than for the σ_2_ subtype, and that BD 1063 also attenuated the locomotor activity induced by this compound [15]. Furthermore, BMY 14802 and MS-377, two known σ_1_ antagonists, inhibited the behavioral sensitization induced by the repeated administration of methamphetamine [1, 196, 205]. As in studies with repeated cocaine administration, it was thought that σ_1_ receptors might play a role in neuronal plasticity after the repeated administration of methamphetamine. Sigma_1_ receptor levels were recently found to be unaltered in rats passively treated with this psychostimulant; however, in rats self-administered with methamphetamine, σ_1_ receptors were upregulated in the rat midbrain, an area involved in learning and reward processes, but not in the cerebellum, frontal cortex, striatum and hippocampus. These observations underscored the role of σ_1_ receptors in neuronal plasticity after the consumption of psychostimulants [191].

The role of σ_1_ receptors in the behavior of other drugs of abuse has also been explored, and it was found that the σ_1_ antagonist BD 1047 was effective against ethanol-induced locomotor stimulation, conditioned place preference, taste aversion and some symptoms of the abstinence syndrome after chronic ethanol consumption [125, 135]. Interestingly, σ_1_ receptor expression was increased in the hippocampus of mice after chronic ethanol consumption. However, both the σ_1_ agonist JO-1784 and the antagonist BD 1047 shared some ameliorating properties against the abstinence syndrome after chronic ethanol consumption [135]. These observations suggest a new pharmacological target for alleviating ethanol addiction and abstinence syndrome after withdrawal, although more behavioral tests should be performed. Interestingly, an association has been suggested between polymorphisms in the σ_1_ receptor gene and alcoholism [139], supporting the role of σ_1_ receptors in chronic ethanol consumption.

## 5 CONCLUSIONS AND PERSPECTIVES

At the present time it seems logical to attribute the neuropharmacological properties of σ_1_ ligands to the neuromodulatory role of σ_1_ receptors. They act as intracellular amplifiers for signal transductions involving InsP_3_ receptors, are clearly able to modulate neurotransmitter systems (mainly through NMDA receptors) and ion channels (such as K^+^ channels), and may play an important role in neuroplasticity processes. Because of this typically modulatory nature of σ_1_ receptors, σ_1_ ligands are usually devoid of effect *per se* under control conditions in many experimental situations. In fact, and in agreement with the modulatory role of σ_1_ receptors, σ_1_ knockout mice do not display any overt phenotype. However, data showed that σ_1_ ligands are highly active when a pharmacological or pathological imbalanced state arises. In addition, and also due to the modulatory role of σ_1_ receptors, the combined administration of σ_1_ receptor ligands and medications with a known therapeutic effect has been shown to improve the effects of the latter (at least in behavioral models of depression and in opioid-mediated analgesia), resulting in the need for lower doses to reach therapeutic concentrations. This synergistic action of σ_1_ ligands and low doses of other known drugs merits further study in additional behavioral models. Of particular interest is the bell-shaped dose-response curve of σ_1_ agonists in *in vitro* experiments, in behavioral tests in which σ_1_ agonists are active (i.e., learning and memory processes, depression and anxiety), and even in some clinical trials. These data strongly suggest that researchers should take hormesis into account in order to design informative experiments or clinical trials with σ_1_ agonists.

In the light of our current knowledge, it seems clear that σ_1_ agonists are promising pharmacological tools against memory and learning disorders, and also against depression and anxiety. Although some previous findings suggest that σ_1_ antagonists might be potentially useful tools against some symptoms of schizophrenia, currently the most promising therapeutic targets for σ_1_ antagonism are nociception and some deleterious effects of certain drugs of abuse (such as cocaine, methamphetamine and ethanol). Importantly, many drugs used routinely in therapeutics show affinity for σ_1_ receptors (see Table **1**), and exert the same effects as other more selective σ_1_ ligands in many behavioral tests and *in vitro* assays. Therefore, the therapeutical properties of these drugs might be due, at least in part, to their interaction with σ_1_ receptors. Interestingly, several drugs have been proved to be effective against diseases (in behavioral animal models) different from those for which they are prescribed in clinical practice, through their interaction with σ_1_ receptors. These findings raise the possibility of new therapeutic applications with drugs routinely used in therapeutics.

## Figures and Tables

**Fig (1):**
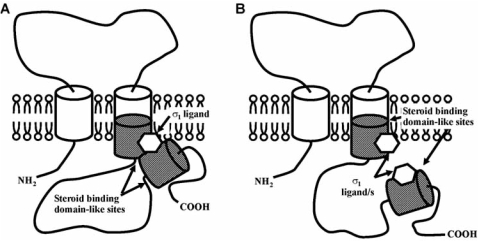
Putative model for σ_1_ receptors proposed by Pal and coworkers [159]. Open cylinders represent the two putative transmembrane domains. Closed cylinders represent the steroid binding domain-like sites and the open hexagon represents a putative σ_1_ ligand. A, Possible spatial arrangement of the ligand binding site involving both steroid binding domain-like sites. B, Alternative model for ligand interaction with the σ_1_ receptor.

**Fig (2):**
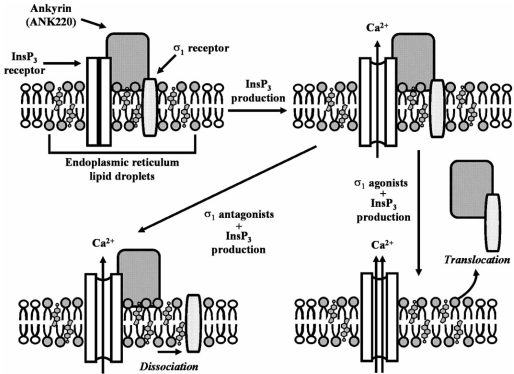
Model of modulation by σ_1_ receptors of InsP3-mediated calcium efflux, proposed by Hayashi and Su [60, 63]. InsP_3_ receptors, ANK220 and σ_1_ receptor form a complex in lipid droplets on the endoplasmic reticulum, which contain moderate amounts of free cholesterol and neutral lipids. In the presence of a σ_1_ agonist, the σ_1_ receptor-ANK220 complex is dissociated from InsP_3_ receptors and translocated. As a result InsP_3_ binding to its receptor increases and Ca2^+^ efflux is enhanced. In the presence of a σ_1_ antagonist, ANK220 remains coupled to InsP_3_ receptor, but σ_1_ receptor is dissociated from the complex, impeding the potentiation of calcium efflux by σ_1_ agonists.

**Table 1:** Pharmacology of some Usual σ_1_ Receptor Ligands

Compound	Subtype Selectivity	Affinity for σ_1_ Site*	Function on σ_1_ Site	Other Activities
**Benzomorphans **				
(+)-Pentazocine	σ_1_[61]	+++ [61]	Agonist [61]	-
(–)-Pentazocine	σ_1_/σ_2_[214]	++ [214]	Agonist [31]	κ_1_ agonist, µ_1_, µ_2_, ligand, low affinity δ, and κ_3_ opioid ligand [31]
(+)-SKF-10,047	σ_1_ [61]	+++ [61]	Agonist [61]	NMDA receptor ligand [61]
**Antipsychotics**				
Chlorpromazine	σ_1_/σ_2_ [108]	++ [61]	? [61]	Dopamine D_2_ antagonist [61]
Haloperidol	σ_1_/σ_2_ [61]	+++ [61]	Antagonist [61]	Dopamine D_2_ and D_3_ antagonist [75]; σ_2_ agonist [121]
Nemonapride	σ_1_/σ_2_? [61]	+++ [61]	? [61]	Dopamine D_2_ antagonist [61]
**Antidepressants**				
Clorgyline	σ_1_ [74]	+++ [74]	Agonist? [9]	Irreversible monoamine oxidase A inhibitor [74]
Fluoxetine	σ_1_ [149]	+ [149]	Agonist [61]	Selective 5-HT reuptake inhibitor [149, 61]
Fluvoxamine	σ_1_ [149]	+++ [149]	Agonist [61]	Selective 5-HT reuptake inhibitor[149, 61]
Imipramine	σ_1_ [149]	++ [149]	Agonist [61]	Monoamine reuptake inhibitor [61]
Sertraline	σ_1_ [149]	++ [149]	Agonist [9]	Selective 5-HT reuptake inhibitor [149]
**Antitussives**				
Carbetapentane	σ_1_/σ_2_ [19]	+++ [19]	Agonist [121]	Muscarinic antagonist [19]
Dextromethorphan	σ_1_ [182]	++ [182]	Agonist [121]	NMDA receptor allosteric antagonist [93]
Dimemorfan	σ_1_/σ_2_ [182]	++ [182]	Agonist [182, 217]	?
**Parkinson’s and/or Alzheimer’s disease**				
Amantadine	?	+ [162]	Agonist? [162]	NMDA antagonist, antiviral properties [25]
Donepezil	σ_1_/σ_2_? [82]	+++? [82]	Agonist [126, 136,137]	Cholinesterase inhibitor [82]
Memantine	?	+ [162]	Agonist? [162]	NMDA antagonist, antiviral properties [25]
**Drugs of abuse**				
Cocaine	σ_1_/σ_2_ [111]	+ [61, 111]	Agonist [61]	Monoamine transporters inhibitor, amongst other actions [175]
MDMA	σ_1_/σ_2_[15]	+ [15]	?	Preferential SERT inhibitor, among other actions [51]
Metamphetamine	σ_1_/σ_2_ [151]	+ [151]	?	Preferential DAT inhibitor, amongst other actions [45]
**Putative endogenous ligands (neurosteroids)**				
DHEAS	σ_1_ [61]	+ [61]	Agonist [61]	GABA_A_ negative modulator [121]
Pregnenolone sulfate	σ_1_ [61]	+ [61]	Agonist [61]	NMDA positive/GABA_A_ negative modulator [121]
Progesterone	σ_1_ [61]	+ [32, 33, 70]	Antagonist [61]	NMDA negative/GABA_A_ positive modulator [121]
**Anticonvulsants**				
Phenytoin (DPH)	σ_1_ [214]	Not applicable	Allosteric Modulator [32, 33, 214]	Delayed rectifier K^+^ channel blocker [152]; T-type Ca^2+^ current inhibitor [202]; Na^+^ current inhibitor [177]
Ropizine	σ_1_ [214]	Not applicable	Allosteric modulator [214]	?
**Other σ drugs**				
BD 737	σ_1_/σ_2_ [65]	+++ [54]	Agonist [54]	-
BD 1008	σ_1_/σ_2_ [61]	+++ [61]	Antagonist [61]	σ_2_ agonist? [120]
BD 1047	σ_1_ [107]	+++ [107]	Antagonist[107]	α adrenoceptor ligand [107]
BD 1063	σ_1_ [107]	+++ [107]	Antagonist [107]	-
BMY 14802	σ_1_/σ_2_ [108]	++ [108]	Antagonist [54]	5-HT_1A_ agonist [106]
DTG	σ_1_/σ_2_ [61]	+++ [61]	? [61]	σ_2_ agonist [121]
Dup 734	σ_1_ [61]	+++ [61]	Antagonist [54]	5-HT_2_ antagonist [200]
Eliprodil (SL-82.0715)	σ_1_/σ_2_ [56]	++ [56]	? [61]	NMDA antagonist,α_1_ adrenoceptor ligand [56]
E-5842	σ_1_ [53]	+++ [53]	Antagonist [54]	Low to moderate affinity for dopamine, 5-HT and glutamate receptors [53]
Haloperidol Metabolite I	σ_1_ [108]	++ [34, 108]	Antagonist [22]	-
Haloperidol Metabolite II	σ_1_/σ_2_ [108]	+++ [34, 108]	Irreversible antagonist [34]	Dopamine D_2_ and D_3_ ligand [75]
4-IBP	σ_1_/σ_2_[77]	+++ [77]	Agonist [9]	Dopamine D_2_ ligand [77]
JO-1784 (Igmesine)	σ_1_ [61]	+++ [61]	Agonist [61]	-
Metaphit	σ_1_/σ_2_ [11]	++ [34]	Irreversible antagonist [11]	Acylator of PCP and σ_2_ binding sites [11]
(+)-MR 200	σ_1_/σ_2_ [173]	+++ [173]	Antagonist [100]	-
MS-377	σ_1_ [61]	+++ [61]	Antagonist [61]	-
NE‑100	σ_1_ [61]	+++ [61]	Antagonist [61]	-
OPC-14523	σ_1_/σ_2_ [61]	+++ [61]	Agonist [54]	Agonist of pre- and post-synaptic 5-HT_1A_ receptors [10]; SERT inhibitor [203]
Panamesine (EMD 57445)	σ_1_/σ_2_? [61]	+++? [61]	Antagonist [54]	One of its metabolites is a dopaminergic antagonist [61]
(+)-3-PPP	σ_1_/σ_2_[64]	++ [32, 33]	Agonist [61]	σ_2_ agonist [121]; NMDA receptor ligand [68]; dopaminergic agonist [61]
PRE 084	σ_1_ [61]	+++ [61]	Agonist [61]	-
Rimcazole (BW-234U)	σ_1_/σ_2_[110]	+ [61]	Antagonist [61]	DAT inhibitor [110]
SA4503	σ_1_ [61]	+++ [61]	Agonist [61]	-
SR 31742A	?	+++ [61]	?	High affinity for C8-C7 sterol isomerase [61]

* K_i_ or K_D_ values:

+++ < 50 nM;

++ < 500 nM;

+ < 10 µM.

? : not studied or unclear at the moment.

- : no other pharmacological target has been described.

**Table 2:** Summary of the Effects of σ_1_ Receptors in Experimental Models of Learning and Memory (see References and Text for Detailed Information)

Involvement of σ_1_ Receptors in Learning and Memory				
Behavioral Assays				
			Effect of σ_1_ Agonists	Effect of σ_1_ Antagonism
Cognitive impairment induced by	Drugs, chemicals or brain lessions	Scopolamine [121, 146]	Improvement	Reversion of the effects of σ_1_ agonists
		Mecamylamine [121]	Improvement	Not tested
		Basal forebrain lesion [121, 146]		
		PCA [115, 116]		
		Dizocilpine [120, 121, 122, 123, 126, 127, 146]	Improvement	Reversion of the effects of σ_1_ agonists
		CO [16, 81, 118, 120, 135]		
	Ageing-related diseases	Aged animals [146]	Improvement	Not tested
		Senescence-accelerated mice [118, 146]	Improvement	Reversion of the effects of σ_1_ agonists
		β_25-35_-amyloid-related peptide (Alzheimer disease-type amnesia) [119, 137, 217]		
	Alterations during pregnancy	Stress [134]	Improvement	Reversion of the effects of σ_1_ agonists
		Cocaine administration [133]		
Cognitive amelioration induced by	Low doses of cocaine [171]		Enhancement	Inhibition
Effects on mechanisms involved in memory and learning impairment or potentiation				
Impairment	Neuronal injury induced by ischemia [16, 81, 118] or β_25-35_-amyloid-related peptide [99]		Neuroprotective effects	Reversion of the effects of σ_1_ agonists^a^
Potentiation	Long-term potentiation [26, 94, 103]		Enhancement	Reversion of the effects of σ_1_ agonists

a Some nonselective σ_1_ antagonists exert neuroprotective effects [reviewed in 118], which may be due to a non-σ_1_-mediated mechanism.

**Table 3:** Summary of the Involvement of σ_1_ Receptors in Depression (see References and Text for Additional Information)

Involvement of σ_1_ Receptors in Depression			
		Effect of σ_1_ Agonists	Effect of σ_1_ Antagonists
Behavioral experimental models	Tail suspension test [207]	Improvement	Reversion of the effects of σ_1_ agonists
	Forced swimming test [121, 126, 185, 208, 210, 218]		
	Olfactory bulbectomy [216]		
Mechanisms associated with antidepressant activity	Firing of serotonergic neurons [8, 203]	Potentiation	Reversion of the effects of σ_1_ agonists
	Neurotrophic actions [198]	Potentiation of growth factor-induced neurite sprouting	
Mechanisms associated with depression	Decrease of NMDA receptor subunit 1 [216]	Reversion	Reversion of the effects of σ_1_ agonists

**Table 4:** Summary of the Involvement of σ_1_ Receptors in Anxiety (see References Cited in the Text for Detailed Information)

Involvement of σ_1_ Receptors on Anxiety			
		Effect of σ_1_ Agonists	Effect of σ_1_ Antagonists
Behavioral experimental models	Conditioned fear stress [79, 80, 153, 211]	Improvement	Reversion of the effects of σ_1_ agonists
	Sexual dysfunction induced by stress [140]		
	Marble-burying behavior test [44]		
	Colonic motor disturbances induced by fear [52]		
Clinical trials (phase-1)	Functional diarrhea [213]	Improvement	Not tested

**Table 5:** Summary of the Involvement of σ_1_ Receptors in Schizophrenia (see References and Text for Detailed Information)

Involvement of σ_1_ Receptors on Schizophrenia				
			Effect of σ_1_ Agonists	Effect of σ_1_ Antagonists
Behavioral experimental models	Dopaminergic function prominently enhanced	Apomorphine-induced climbing [53, 187, 195, 201]	Not tested	Inhibition
		Amphetamine-induced locomotor activity [53, 164, 176, 187]		
		Behavioral sensitization induced by repeated administration of psychostimulants [1, 196, 205, 206, 221]		
	Glutamergic function prominently disturbed	PCP-induced stereotyped behaviors [63, 187, 195]	Not tested	Inhibition
		Dizocilpine-induced hyperlocomotion in monoamine depleted mice [157]	Enhancement	Reversion of the effects of σ_1_ agonists
		PCP-induced cognitive deficits [58]	Improvement	Reversion of the effects of σ_1_ agonists
Clinical trials	Only with BMY 14802, eliprodil and panamesine [61]		Not tested	Inconclusive results

**Table 6:** Summary of the Involvement of σ_1_ Receptors in Analgesia (see Text and References for Detailed Information, as Administration Routes of Drugs)

Involvement of σ_1_ Receptors on Analgesia			
	Behavioral Experimental Models	Effect of σ_1_ Agonists	Effect of σ_1_ Antagonism
Modulation of opioid analgesia	Tail-flick test [28-30, 87, 100, 130, 161, 173]	Inhibition	Enhancement
	Acetic acid-induced writhings [66, 67]	Inactive (very low doses tested)^a^	Inactive (very low doses tested)^a^
Pain modulation in the absence of opioid drugs	Tail-flick test [22, 28-30, 100, 130, 161]	Inactive	Inactive
	Acetic acid-induced writhings [66, 67]	Inactive(very low doses tested)^a^	Inactive(very low doses tested)^a^
	Nociceptive flexor response test [204]	Nociception	Reversion of the effects of σ_1_ agonists
	Formalin-induced pain [22, 23, 86]	Reversion of the effects of σ_1_ antagonists	Antinociception
	Plantar test [85]	Thermal hyperalgesia^b^	Reversion of the effects of σ_1_ agonists
	von Frey test [85]	Mechanical allodynia^b^	Reversion of the effects of σ_1_ agonists

a Additional experiments using higher doses of σ_1_ ligands should be performed.

b Selective σ_1_ agonists should be tested.

**Table 7:** Summary of the Involvement of σ_1_ Receptors in the Behavioral Effects Induced by Cocaine (see References and Text for Detailed Information)

Involvement of σ_1_ Receptors in Cocaine-Induced Behavioral Effects					
			Behavioral Experimental Model	Effect of σ_1_ Agonists	Effect of σ_1_ Antagonism
Acute effects of cocaine	Psychostimulant effects		Locomotor activity [37, 95, 111, 109, 112, 124]	Potentiation	Inhibition
	Toxicity		Convulsions [37, 109, 111, 112, 113, 124]		
			Lethality [37, 112, 113, 124]		
Repeated administration of cocaine	Self-reported effects of cocaine		Drug discrimination test [83, 95, 221]	Not tested	Slight or no effect
	Nervous system plasticity		Locomotor sensitization [206, 221]	Not tested	Inhibition
	Rewarding properties	During addictive behavior	Conditioned place preference [168, 169]	Not tested	Inhibition
			Self-administration [101]	Not tested	No effect
		After extinction of addictive behavior	Conditioned place preference after priming injection of drugs [170]	Reactivation	Inhibition
			Discriminative stimulus associated with cocaine availability for self-administration [101]	Not tested	Inhibition

## References

[1] Akiyama K, Kanzaki A, Tsuchida K, Ujike H (1994). Methamphetamine-induced behavioral sensitization and its implications for relapse of schizophrenia. Schizophr. Res.

[2] Alonso G, Phan V, Guillemain I, Saunier M, Legrand A, Anoal M, Maurice T (2000). Immunocytochemical localization of the sigma_1_ receptor in the adult rat central nervous system. Neuroscience.

[3] Aydar E, Palmer CP, Klyachko VA, Jackson MB (2002). The sigma receptor as a ligand-regulated auxiliary potassium channel subunit. Neuron.

[4] Bartus RT (2000). On neurodegenerative diseases, models, and treatment strategies: lessons learned and lessons forgotten a generation following the cholinergic hypothesis. Exp. Neurol.

[5] Baulieu EE (1998). Neurosteroids: a novel function of the brain. Psychoneuroendocrinology.

[6] Bergeron R, de Montigny C, Debonnel G (1996). Potentiation of neuronal NMDA response induced by dehydroepiandrosterone and its suppression by progesterone: effects mediated *via* sigma receptors. J. Neurosci.

[7] Bergeron R, de Montigny C, Debonnel G (1999). Pregnancy reduces brain sigma receptor function. Br. J. Pharmacol.

[8] Bermack JE, Debonnel G (2001). Modulation of serotonergic neurotransmission by short- and long-term treatments with sigma ligands. Br. J. Pharmacol.

[9] Bermack JE, Debonnel G (2005). The role of sigma receptors in depression. J. Pharmacol. Sci.

[10] Bermack JE, Debonnel G (2007). Effects of OPC-14523, a combined sigma and 5-HT_1a_ ligand, on pre- and post-synaptic 5-HT_1a_ receptors. J. Psychopharmacol.

[11] Bluth LS, Rice KC, Jacobson AE, Bowen WD (1989). Acylation of σ receptors by Metaphit, an isothiocyanate derivative of phencyclidine. Eur. J. Pharmacol.

[12] Bowen WD, Hellewell SB, McGarry KA (1989). Evidence for a multi-site model of the rat brain σ receptor. Eur. J. Pharmacol.

[13] Bowen WD, Moses EL, Tolentino PJ, Walker JM (1990). Metabolites of haloperidol display preferential activity at σ receptors compared to dopamine D-2 receptors. Eur. J. Pharmacol.

[14] Bowen WD (2000). Sigma receptors: recent advances and new clinical potentials. Pharm. Acta Helv.

[15] Brammer MK, Gilmore DL, Matsumoto RR (2006). Interactions between 3,4-methylenedioxymethamphetamine and σ_1_ receptors. Eur. J. Pharmacol.

[16] Bucolo C, Marrazzo A, Ronsisvalle S, Ronsisvalle G, Cuzzocrea S, Mazzon E, Caputi A, Drago F (2006). A novel adamantane derivative attenuates retinal ischemia-reperfusion damage in the rat retina through σ_1_ receptors. Eur. J. Pharmacol.

[17] Cagnotto A, Bastone A, Mennini T (1994). [^3^H](+)-pentazocine binding to rat brain sigma_1_ receptors. Eur. J. Pharmacol.

[18] Calabrese EJ, Baldwin LA (2003). Hormesis: the dose-response revolution. Annu. Rev. Pharmacol. Toxicol.

[19] Calderon SN, Izenwasser S, Heller B, Gutkind JS, Mattson MV, Su TP, Newman AH (1994). Novel 1-phenylcycloalkane-carboxylic acid derivatives are potent and selective σ_1_ ligands. J. Med. Chem.

[20] Campana G, Bucolo C, Murari G, Spampinato S (2002). Ocular hypotensive action of topical flunarizine in the rabbit: role of σ_1_ recognition sites. J. Pharmacol. Exp. Ther.

[21] Castner SA, Goldman-Rakic PS, Williams GV (2004). Animal models of working memory: insights for targeting cognitive dysfunction in schizophrenia. Psychopharmacology (Berl).

[22] Cendan CM, Pujalte JM, Portillo-Salido E, Baeyens JM (2005). Antinociceptive effects of haloperidol and its metabolites in the formalin test in mice. Psychopharmacology (Berl).

[23] Cendan CM, Pujalte JM, Portillo-Salido E, Montoliu L, Baeyens JM (2005). Formalin-induced pain is reduced in σ_1_ receptor knockout mice. Eur. J. Pharmacol.

[24] Chavez-Noriega LE, Marino MJ, Schaffhauser H, Campbell HUC, Conn PJ (2005). Novel potential therapeutics for schizophrenia: focus on the modulation of metabotropic glutamate receptor function. Curr. Neuropharmacol.

[25] Chen HS, Lipton SA (2006). The chemical biology of clinically tolerated NMDA receptor antagonists. J. Neurochem.

[26] Chen L, Dai XN, Sokabe M (2006). Chronic administration of dehydroepiandrosterone sulfate (DHEAS) primes for facilitated induction of long-term potentiation *via* sigma 1 (σ_1_) receptor: optical imaging study in rat hippocampal slices. Neuropharmacology.

[27] Chen Y, Hajipour AR, Sievert MK, Arbabian M, Ruoho AE (2007). Characterization of the cocaine binding site on the sigma-1 receptor. Biochemistry.

[28] Chien CC, Pasternak GW (1993). Functional antagonism of morphine analgesia by (+)-pentazocine: evidence for an anti-opioid σ_1_ system. Eur. J. Pharmacol.

[29] Chien CC, Pasternak GW (1994). Selective antagonism of opioid analgesia by a *sigma* system. J. Pharmacol. Exp. Ther.

[30] Chien CC, Pasternak GW (1995). Sigma antagonists potentiate opioid analgesia in rats. Neurosci. Lett.

[31] Chien CC, Pasternak GW (1995). (-)-Pentazocine analgesia in mice: interactions with a σ receptor system. Eur. J. Pharmacol.

[32] Cobos EJ, Baeyens JM, Del Pozo E (2005). Phenytoin differentially modulates the affinity of agonist and antagonist ligands for σ_1_ receptors of guinea pig brain. Synapse.

[33] Cobos EJ, Lucena G, Baeyens JM, Del Pozo E (2006). Differences in the allosteric modulation by phenytoin of the binding properties of the σ_1_ligands [^3^H](+)-pentazocine and [^3^H]NE-100. Synapse.

[34] Cobos EJ, del Pozo E, Baeyens JM (2007). Irreversible blockade of sigma-1 receptors by haloperidol and its metabolites in guinea pig brain and SH-SY5Y human neuroblastoma cells. J. Neurochem.

[35] Collina S, Loddo G, Urbano M, Linati L, Callegari A, Ortuso F, Alcaro S, Laggner C, Langer T, Prezzavento O, Ronsisvalle G, Azzolina O (2007). Design, synthesis, and SAR analysis of novel selective *σ*_1_ ligands. Bioorg. Med. Chem.

[36] Cormaci G, Mori T, Hayashi T, Su TP (2007). Protein kinase A activation down-regulates, whereas extracellular signal-regulated kinase activation up-regulates σ-1 receptors in B-104 cells: Implication for neuroplasticity. J. Pharmacol. Exp. Ther.

[37] Daniels A, Ayala E, Chen W, Coop A, Matsumoto RR (2006). N-[2-(*m*-methoxyphenyl)ethyl]-*N*-ethyl-2-(1-pyrrolidinyl) ethylamine (UMB 116) is a novel antagonist for cocaine-induced effects. Eur. J. Pharmacol.

[38] DeHaven-Hudkins DL, Lanyon LF, Ford-Rice FY, Ator MA (1994). σ recognition sites in brain and peripheral tissues. Characterization and effects of cytochrome P450 inhibitors. Biochem. Pharmacol.

[39] Delgado PL, Moreno FA (2000). Role of norepinephrine in depression. J. Clin. Psychiatry.

[40] Depatie L, Lal S (2001). Apomorphine and the dopamine hypothesis of schizophrenia: a dilemma?. J. Psychiatry. Neurosci.

[41] Dhir A, Kulkarni SK (2007). Involvement of sigma-1 receptor modulation in the antidepressant action of venlafaxine. Neurosci. Lett.

[42] Dong LY, Cheng ZX, Fu YM, Wang ZM, Zhu YH, Sun JL, Dong Y, Zheng P (2007). Neurosteroid dehydroepiandrosterone sulfate enhances spontaneous glutamate release in rat prelimbic cortex through activation of dopamine D1 and sigma-1 receptor. Neuropharmacology.

[43] Dussossoy D, Carayon P, Belugou S, Feraut D, Bord A, Goubet C, Roque C, Vidal H, Combes T, Loison G, Casellas P (1999). Colocalization of sterol isomerase and sigma_1_ receptor at endoplasmic reticulum and nuclear envelope level. Eur. J. Biochem.

[44] Egashira N, Harada S, Okuno R, Matsushita M, Nishimura R, Mishima K, Iwasaki K, Orito K, Fujiwara M (2007). Involvement of the sigma_1_ receptor in inhibiting activity of fluvoxamine on marble-burying behavior: comparison with paroxetine. Eur. J. Pharmacol.

[45] Fleckenstein AE, Volz TJ, Riddle EL, Gibb JW, Hanson GR (2007). New insights into the mechanism of action of amphetamines. Annu. Rev. Pharmacol. Toxicol.

[46] Gardner B, Zhu LX, Roth MD, Tashkin DP, Dubinett SM, Sharma S (2004). Cocaine modulates cytokine and enhances tumor growth through sigma receptors. J. Neuroimmunol.

[47] Garrone B, Magnani M, Pinza M, Polenzani L (2000). Effects of trazodone on neurotransmitter release from rat mossy fibre cerebellar synaptosomes. Eur. J. Pharmacol.

[48] Gear RW, Lee JS, Miaskowski C, Gordon NC, Paul SM, Levine JD (2006). Neuroleptics antagonize nalbuphine antianalgesia. J. Pain.

[49] Gekker G, Hu S, Sheng WS, Rock RB, Lokensgard JR, Peterson PK (2006). Cocaine-induced HIV-1 expression in microglia involves sigma-1 receptors and transforming growth factor-β1. Int. Immunopharmacol.

[50] Gewirtz GR, Gorman JM, Volavka J, Macaluso J, Gribkoff G, Taylor DP, Borison R (1994). BMY 14802, a sigma receptor ligand for the treatment of schizophrenia. Neuropsychopharmacology.

[51] Green AR, Mechan AO, Elliott JM, O'Shea E, Colado MI (2003). The pharmacology and clinical pharmacology of 3,4-methylenedioxymethamphetamine (MDMA, "ecstasy"). Pharmacol. Rev.

[52] Gue M, Junien JL, Del Rio C, Bueno L (1992). Neuropeptide Y and sigma ligand (JO 1784) suppress stress-induced colonic motor disturbances in rats through *sigma* and cholecystokinin receptors. J. Pharmacol. Exp. Ther.

[53] Guitart X, Codony X, Ballarín M, Dordal A, Farré AJ (1998). E-5842: a new potent and preferential σ ligand: preclinical pharmacological profile. CNS Drug Rev.

[54] Guitart X, Codony X, Monroy X (2004). Sigma receptors: biology and therapeutic potential. Psychopharmacology (Berl).

[55] Hanner M, Moebius FF, Flandorfer A, Knaus HG, Striessnig J, Kempner E, Glossmann H (1996). Purification, molecular cloning, and expression of the mammalian sigma_1_-binding site. Proc. Natl. Acad. Sci. USA.

[56] Hashimoto K, London ED (1995). Interactions of *erythro*-ifenprodil, *threo*-ifenprodil, *erythro*-iodoifenprodil, and eliprodil with subtypes of *σ* receptors. Eur. J. Pharmacol.

[57] Hashimoto K, Fujita Y, Shimizu E, Iyo M (2005). Phencyclidine-induced cognitive deficits in mice are improved by subsequent subchronic administration of clozapine, but not haloperidol. Eur. J. Pharmacol.

[58] Hashimoto K, Fujita Y, Iyo M (2006). Phencyclidine-Induced Cognitive Deficits in Mice are Improved by Subsequent Subchronic Administration of Fluvoxamine: role of sigma-1 receptors. Neuropsychopharmacology.

[59] Hayashi T, Maurice T, Su TP (2000). Ca^2+^ signaling *via* σ_1_-receptors: novel regulatory mechanism affecting intracellular Ca^2+^ concentration. J. Pharmacol. Exp. Ther.

[60] Hayashi T, Su TP (2001). Regulating ankyrin dynamics: Roles of sigma-1 receptors. Proc. Natl. Acad. Sci. USA.

[61] Hayashi T, Su TP (2004). σ-1 receptor ligands: potential in the treatment of neuropsychiatric disorders. CNS. Drugs.

[62] Hayashi T, Su TP (2005). The potential role of sigma-1 receptors in lipid transport and lipid raft reconstitution in the brain: implication for drug abuse. Life Sci.

[63] Hayashi T, Su TP (2005). The Sigma Receptor: Evolution of the Concept in Neuropsychopharmacology. Curr. Neuropharmacol.

[64] Hellewell SB, Bowen WD (1990). A sigma-like binding site in rat pheochromocytoma (PC12) cells: decreased affinity for (+)-benzomorphans and lower molecular weight suggest a different sigma receptor form from that of guinea pig brain. Brain Res.

[65] Hellewell SB, Bruce A, Feinstein G, Orringer J, Williams W, Bowen WD (1994). Rat liver and kidney contain high densities of σ_1_ and σ_2_ receptors: characterization by ligand binding and photoaffinity labeling. Eur. J. Pharmacol.

[66] Hiramatsu M, Hoshino T, Kameyama T, Nabeshima T (2002). Involvement of κ-opioid and σ receptors in short-term memory in mice. Eur. J. Pharmacol.

[67] Hiramatsu M, Hoshino T (2004). Involvement of κ-opioid receptors and σ receptors in memory function demonstrated using an antisense strategy. Brain Res.

[68] Hofner G, Wanner KT (2000). [^3^H]ifenprodil binding to NMDA receptors in porcine hippocampal brain membranes. Eur. J. Pharmacol.

[69] Hong W, Werling LL (2002). Binding of σ receptor ligands and their effects on muscarine-induced Ca^2+^ changes in SH-SY5Y cells. Eur. J. Pharmacol.

[70] Hong W, Nuwayhid SJ, Werling LL (2004). Modulation of bradykinin-induced calcium changes in SH-SY5Y cells by neurosteroids and sigma receptor ligands *via* a shared mechanism. Synapse.

[71] Horan B, Gifford AN, Matsuno K, Mita S, Ashby CR Jr (2002). Effect of SA4503 on the electrically evoked release of ^3^H-acetylcholine from striatal and hippocampal rat brain slices. Synapse.

[72] Introini-Collison IB, McGaugh JL (1989). Cocaine enhances memory storage in mice. Psychopharmacology (Berl).

[73] Ishikawa M, Ishiwata K, Ishii K, Kimura Y, Sakata M, Naganawa M, Oda K, Miyatake R, Fujisaki M, Shimizu E, Shirayama Y, Iyo M, Hashimoto K (2007). High occupancy of sigma-1 receptors in the human brain after single oral administration of fluvoxamine: A positron emission tomography study using [^11^C]SA4503. Biol. Psychiatry.

[74] Itzhak Y, Stein I, Zhang SH, Kassim CO, Cristante D (1991). Binding of σ-ligands to C57BL/6 mouse brain membranes: effects of monoamine oxidase inhibitors and subcellular distribution studies suggest the existence of σ-receptor subtypes. J. Pharmacol. Exp. Ther.

[75] Jaen JC, Caprathe BW, Pugsley TA, Wise LD, Akunne H (1993). Evaluation of the effects of the enantiomers of reduced haloperidol, azaperol, and related 4-amino-1-arylbutanols on dopamine and σ receptors. J. Med. Chem.

[76] Jiang G, Mysona B, Dun Y, Gnana-Prakasam JP, Pabla N, Li W, Dong Z, Ganapathy V, Smith SB (2006). Expression, subcellular localization, and regulation of sigma receptor in retinal muller cells. Invest. Ophthalmol. Vis. Sci.

[77] John CS, Vilner BJ, Bowen WD (1994). Synthesis and characterization of [^125^I]-N-(N-benzylpiperidin-4-yl)-4- iodobenzamide, a new σ receptor radiopharmaceutical: high-affinity binding to MCF-7 breast tumor cells. J. Med. Chem.

[78] Kalasinsky KS, Bosy TZ, Schmunk GA, Ang L, Adams V, Gore SB, Smialek J, Furukawa Y, Guttman M, Kish SJ (2000). Regional distribution of cocaine in postmortem brain of chronic human cocaine users. J. Forensic Sci.

[79] Kamei H, Kameyama T, Nabeshima T (1996). (+)-SKF-10,047 and dextromethorphan ameliorate conditioned fear stress through the activation of phenytoin-regulated σ_1_ sites. Eur. J. Pharmacol.

[80] Kamei H, Noda Y, Kameyama T, Nabeshima T (1997). Role of (+)-SKF-10,047-sensitive sub-population of σ_1_ receptors in amelioration of conditioned fear stress in rats: association with mesolimbic dopaminergic systems. Eur. J. Pharmacol.

[81] Katnik C, Guerrero WR, Pennypacker KR, Herrera Y, Cuevas J (2006). Sigma-1 receptor activation prevents intracellular calcium dysregulation in cortical neurons during *in vitro* ischemia. J. Pharmacol. Exp. Ther.

[82] Kato K, Hayako H, Ishihara Y, Marui S, Iwane M, Miyamoto M (1999). TAK-147, an acetylcholinesterase inhibitor, increases choline acetyltransferase activity in cultured rat septal cholinergic neurons. Neurosci. Lett.

[83] Katz JL, Libby TA, Kopajtic T, Husbands SM, Newman AH (2003). Behavioral effects of rimcazole analogues alone and in combination with cocaine. Eur. J. Pharmacol.

[84] Kekuda R, Prasad PD, Fei YJ, Leibach FH, Ganapathy V (1996). Cloning and functional expression of the human type 1 sigma receptor (hSigmaR1). Biochem. Biophys. Res. Commun.

[85] Kibaly C, Meyer L, Patte-Mensah C, Mensah-Nyagan AG (2008). Biochemical and functional evidence for the control of pain mechanisms by dehydroepiandrosterone endogenously synthesized in the spinal cord. FASEB J.

[86] Kim HW, Kwon YB, Roh DH, Yoon SY, Han HJ, Kim KW, Beitz AJ, Lee JH (2006). Intrathecal treatment with σ_1_ receptor antagonists reduces formalin-induced phosphorylation of NMDA receptor subunit 1 and the second phase of formalin test in mice. Br. J. Pharmacol.

[87] King M, Pan YX, Mei J, Chang A, Xu J, Pasternak GW (1997). Enhanced κ-opioid receptor-mediated analgesia by antisense targeting the σ_1_ receptor. Eur. J. Pharmacol.

[88] Kitaichi K, Chabot JG, Moebius FF, Flandorfer A, Glossmann H, Quirion R (2000). Expression of the purported sigma_1_ (σ_1_) receptor in the mammalian brain and its possible relevance in deficits induced by antagonism of the NMDA receptor complex as revealed using an antisense strategy. J. Chem. Neuroanat.

[89] Kofman O (2002). The role of prenatal stress in the etiology of developmental behavioural disorders. Neurosci. Biobehav. Rev.

[90] Kurtz MM (2005). Neurocognitive impairment across the lifespan in schizophrenia: an update. Schizophr. Res.

[91] Langa F, Codony X, Tovar V, Lavado A, Gimenez E, Cozar P, Cantero M, Dordal A, Hernandez E, Perez R, Monroy X, Zamanillo D, Guitart X, Montoliu L (2003). Generation and phenotypic analysis of sigma receptor type I (σ 1) knockout mice. Eur. J. Neurosci.

[92] Law AJ, Deakin JF (2001). Asymmetrical reductions of hippocampal NMDAR1 glutamate receptor mRNA in the psychoses. Neuroreport.

[93] LePage KT, Ishmael JE, Low CM, Traynelis SF, Murray TF (2005). Differential binding properties of [^3^H]dextrorphan and [^3^H]MK-801 in heterologously expressed NMDA receptors. Neuropharmacology.

[94] Li Z, Zhou R, Cui S, Xie G, Cai W, Sokabe M, Chen L (2006). Dehydroepiandrosterone sulfate prevents ischemia-induced impairment of long-term potentiation in rat hippocampal CA1 by up-regulating tyrosine phosphorylation of NMDA receptor. Neuropharmacology.

[95] Liu X, Banister SD, Christie MJ, Banati R, Meikle S, Coster MJ, Kassiou M (2007). Trishomocubanes: novel σ ligands modulate cocaine-induced behavioural effects. Eur. J. Pharmacol.

[96] Liu Y, Chen GD, Lerner MR, Brackett DJ, Matsumoto RR (2005). Cocaine up-regulates Fra-2 and σ-1 receptor gene and protein expression in brain regions involved in addiction and reward. J. Pharmacol. Exp. Ther.

[97] Lupardus PJ, Wilke RA, Aydar E, Palmer CP, Chen Y, Ruoho AE, Jackson MB (2000). Membrane-delimited coupling between sigma receptors and K^+^ channels in rat neurohypophysial terminals requires neither G-protein nor ATP. J. Physiol.

[98] Marrazzo A, Prezzavento O, Pappalardo MS, Bousquet E, Iadanza M, Pike VW, Ronsisvalle G (2002). Synthesis of (+)- and (-)-cis-2-[(1-adamantylamino)-methyl]-1-phenylcyclopropane derivatives as high affinity probes for σ_1_ and σ_2_ binding sites. Farmaco.

[99] Marrazzo A, Caraci F, Salinaro ET, Su TP, Copani A, Ronsisvalle G (2005). Neuroprotective effects of sigma-1 receptor agonists against beta-amyloid-induced toxicity. Neuroreport.

[100] Marrazzo A, Parenti C, Scavo V, Ronsisvalle S, Scoto GM, Ronsisvalle G (2006). *In vivo* evaluation of (+)-MR200 as a new selective sigma ligand modulating MOP, DOP and KOP supraspinal analgesia. Life Sci.

[101] Martin-Fardon R, Maurice T, Aujla H, Bowen WD, Weiss F (2007). Differential effects of σ_1_ receptor blockade on self-administration and conditioned reinstatement motivated by cocaine vs natural reward. Neuropsychopharmacology.

[102] Martin WR, Eades CG, Thompson JA, Huppler RE, Gilbert PE (1976). The effects of morphine- and nalorphine- like drugs in the nondependent and morphine-dependent chronic spinal dog. J. Pharmacol. Exp. Ther.

[103] Martina M, Turcotte ME, Halman S, Bergeron R (2007). The sigma-1 receptor modulates NMDA receptor synaptic transmission and plasticity *via* SK channels in rat hippocampus. J. Physiol.

[104] Maruo J, Yoshida A, Shimohira I, Matsuno K, Mita S, Ueda H (2000). Binding of [^35^S]GTPγS stimulated by (+)-pentazocine sigma receptor agonist, is abundant in the guinea pig spleen. Life Sci.

[105] Mateo Y, Budygin EA, John CE, Jones SR (2004). Role of serotonin in cocaine effects in mice with reduced dopamine transporter function. Proc. Natl. Acad. Sci. USA.

[106] Matos FF, Korpinen C, Yocca FD (1996). 5-HT_1A_ receptor agonist effects of BMY-14802 on serotonin release in dorsal raphe and hippocampus. Eur. J. Pharmacol.

[107] Matsumoto RR, Bowen WD, Tom MA, Vo VN, Truong DD, De Costa BR (1995). Characterization of two novel σ receptor ligands: antidystonic effects in rats suggest σ receptor antagonism. Eur. J. Pharmacol.

[108] Matsumoto RR, Pouw B (2000). Correlation between neuroleptic binding to σ_1_ and σ_2_ receptors and acute dystonic reactions. Eur. J. Pharmacol.

[109] Matsumoto RR, McCracken KA, Friedman MJ, Pouw B, De Costa BR, Bowen WD (2001). Conformationally restricted analogs of BD1008 and an antisense oligodeoxynucleotide targeting σ_1_ receptors produce anti-cocaine effects in mice. Eur. J. Pharmacol.

[110] Matsumoto RR, Hewett KL, Pouw B, Bowen WD, Husbands SM, Cao JJ, Hauck NA (2001). Rimcazole analogs attenuate the convulsive effects of cocaine: correlation with binding to sigma receptors rather than dopamine transporters. Neuropharmacology.

[111] Matsumoto RR, McCracken KA, Pouw B, Zhang Y, Bowen WD (2002). Involvement of sigma receptors in the behavioral effects of cocaine: evidence from novel ligands and antisense oligodeoxynucleotides. Neuropharmacology.

[112] Matsumoto RR, Liu Y, Lerner M, Howard EW, Brackett DJ (2003). σ receptors: potential medications development target for anti-cocaine agents. Eur. J. Pharmacol.

[113] Matsumoto RR, Gilmore DL, Pouw B, Bowen WD, Williams W, Kausar A, Coop A (2004). Novel analogs of the σ receptor ligand BD1008 attenuate cocaine-induced toxicity in mice. Eur. J. Pharmacol.

[114] Matsumoto RR, Pouw B, Mack AL, Daniels A, Coop A (2007). Effects of UMB24 and (+/-)-SM 21, putative σ_2_-preferring antagonists, on behavioral toxic and stimulant effects of cocaine in mice. Pharmacol. Biochem. Behav.

[115] Matsuno K, Senda T, Matsunaga K, Mita S, Kaneto H (1993). Similar ameliorating effects of benzomorphans and 5-HT_2_ antagonists on drug-induced impairment of passive avoidance response in mice: comparison with acetylcholinesterase inhibitors. Psychopharmacology (Berl).

[116] Matsuno K, Senda T, Matsunaga K, Mita S (1994). Ameliorating effects of σ receptor ligands on the impairment of passive avoidance tasks in mice: involvement in the central acetylcholinergic system. Eur. J. Pharmacol.

[117] Maurice T, Roman FJ, Privat A (1996). Modulation by neurosteroids of the *in vivo* (+)-[^3^H]SKF-10,047 binding to σ_1_ receptors in the mouse forebrain. J. Neurosci. Res.

[118] Maurice T, Lockhart BP (1997). Neuroprotective and anti-amnesic potentials of sigma (σ) receptor ligands. Prog. Neuropsychopharmacol. Biol. Psychiatry.

[119] Maurice T, Su TP, Privat A (1998). Sigma_1_ (σ_1_) receptor agonists and neurosteroids attenuate B_25-35_-amyloid peptide-induced amnesia in mice through a common mechanism. Neuroscience.

[120] Maurice T, Phan VL, Urani A, Kamei H, Noda Y, Nabeshima T (1999). Neuroactive neurosteroids as endogenous effectors for the sigma_1_ (σ_1_) receptor: pharmacological evidence and therapeutic opportunities. Jpn. J. Pharmacol.

[121] Maurice T, Urani A, Phan VL, Romieu P (2001). The interaction between neuroactive steroids and the σ_1_ receptor function: behavioral consequences and therapeutic opportunities. Brain Res. Brain Res. Rev.

[122] Maurice T, Phan VL, Urani A, Guillemain I (2001). Differential involvement of the sigma_1_ (*σ_1_*) receptor in the anti-amnesic effect of neuroactive steroids, as demonstrated using an *in vivo* antisense strategy in the mouse. Br. J. Pharmacol.

[123] Maurice T, Phan VL, Privat A (2001). The anti-amnesic effects of sigma_1_ (*σ*_1_) receptor agonists confirmed by *in vivo* antisense strategy in the mouse. Brain Res.

[124] Maurice T, Martin-Fardon R, Romieu P, Matsumoto RR (2002). Sigma_1_ (σ_1_) receptor antagonists represent a new strategy against cocaine addiction and toxicity. Neurosci. Biobehav. Rev.

[125] Maurice T, Casalino M, Lacroix M, Romieu P (2003). Involvement of the sigma_1_ receptor in the motivational effects of ethanol in mice. Pharmacol. Biochem. Behav.

[126] Maurice T, Meunier J, Feng B, Ieni J, Monaghan DT (2006). Interaction with *σ*_1_ protein, but not N-methyl-D-aspartate receptor, is involved in the pharmacological activity of donepezil. J. Pharmacol. Exp. Ther.

[127] Maurice T, Gregoire C, Espallergues J (2006). Neuro(active) steroids actions at the neuromodulatory sigma_1_ (σ_1_) receptor: biochemical and physiological evidences, consequences in neuroprotection. Pharmacol. Biochem. Behav.

[128] Mavlyutov TA, Ruoho AE (2007). Ligand-dependent localization and intracellular stability of sigma-1 receptors in CHO-K1 cells. J. Mol. Signal.

[129] McCracken KA, Bowen WD, De Costa BR, Matsumoto RR (1999). Two novel σ receptor ligands, BD1047 and LR172, attenuate cocaine-induced toxicity and locomotor activity. Eur. J. Pharmacol.

[130] Mei J, Pasternak GW (2002). σ_1_ receptor modulation of opioid analgesia in the mouse. J. Pharmacol. Exp. Ther.

[131] Mei J, Pasternak GW (2007). Modulation of brainstem opiate analgesia in the rat by *σ*_1_ receptors: a microinjection study. J. Pharmacol. Exp. Ther.

[132] Mennini T, Gobbi M (2004). The antidepressant mechanism of Hypericum perforatum. Life Sci.

[133] Meunier J, Maurice T (2004). Beneficial effects of the sigma_1_ receptor agonists igmesine and dehydroepiandrosterone against learning impairments in rats prenatally exposed to cocaine. Neurotoxicol. Teratol.

[134] Meunier J, Gue M, Recasens M, Maurice T (2004). Attenuation by a sigma_1_ (*σ*_1_) receptor agonist of the learning and memory deficits induced by a prenatal restraint stress in juvenile rats. Br. J. Pharmacol.

[135] Meunier J, Ieni J, Maurice T (2006). Antiamnesic and neuroprotective effects of donepezil against learning impairments induced in mice by exposure to carbon monoxide gas. J. Pharmacol. Exp. Ther.

[136] Meunier J, Demeilliers B, Celerier A, Maurice T (2006). Compensatory effect by sigma_1_ (σ_1_) receptor stimulation during alcohol withdrawal in mice performing an object recognition task. Behav. Brain. Res.

[137] Meunier J, Ieni J, Maurice T (2006). The anti-amnesic and neuroprotective effects of donepezil against amyloid β_25-35_ peptide-induced toxicity in mice involve an interaction with the σ_1_ receptor. Br. J. Pharmacol.

[138] Meyer DA, Carta M, Partridge LD, Covey DF, Valenzuela CF (2002). Neurosteroids enhance spontaneous glutamate release in hippocampal neurons.Possible role of metabotropic *σ_1_*-like receptors. J. Biol. Chem.

[139] Miyatake R, Furukawa A, Matsushita S, Higuchi S, Suwaki H (2004). Functional polymorphisms in the sigma_1_ receptor gene associated with alcoholism. Biol. Psychiatry.

[140] Mizuno T, Yotsuyanagi S, Nagasaka Y, Namiki M (2006). Dehydroepiandrosterone alleviates copulatory disorder induced by social stress in male rats. J. Sex. Med.

[141] Moebius FF, Reiter RJ, Hanner M, Glossmann H (1997). High affinity of sigma_1_-binding sites for sterol isomerization inhibitors: evidence for a pharmacological relationship with the yeast sterol C_8_-C_7_ isomerase. Br. J. Pharmacol.

[142] Monassier L, Manoury B, Bellocq C, Weissenburger J, Greney H, Zimmermann D, Ehrhardt JD, Jaillon P, Baro I, Bousquet P (2007). σ_2_-receptor ligand-mediated inhibition of inwardly rectifying K^+^ channels in the heart. J. Pharmacol. Exp. Ther.

[143] Monnet FP, Debonnel G, Bergeron R, Gronier B, de Montigny C (1994). The effects of sigma ligands and of neuropeptide Y on N-methyl-D-aspartate-induced neuronal activation of CA_3_ dorsal hippocampus neurones are differentially affected by pertussin toxin. Br. J. Pharmacol.

[144] Monnet FP, Morin-Surun MP, Leger J, Combettes L (2003). Protein kinase C-dependent potentiation of intracellular calcium influx by σ_1_ receptor agonists in rat hippocampal neurons. J. Pharmacol. Exp. Ther.

[145] Monnet FP (2005). Sigma-1 receptor as regulator of neuronal intracellular Ca^2+^: clinical and therapeutic relevance. Biol. Cell.

[146] Monnet FP, Maurice T (2006). The sigma_1_ protein as a target for the non-genomic effects of neuro(active)steroids: molecular, physiological, and behavioral aspects. J. Pharmacol. Sci.

[147] Morin-Surun MP, Collin T, Denavit-Saubie M, Baulieu EE, Monnet FP (1999). Intracellular *σ_1_* receptor modulates phospholipase C and protein kinase C activities in the brainstem. Proc. Natl. Acad. Sci. USA.

[148] Mtchedlishvili Z, Kapur J (2003). A presynaptic action of the neurosteroid pregnenolone sulfate on GABAergic synaptic transmission. Mol. Pharmacol.

[149] Narita N, Hashimoto K, Tomitaka S, Minabe Y (1996). Interactions of selective serotonin reuptake inhibitors with subtypes of σ receptors in rat brain. Eur. J. Pharmacol.

[150] Nestler EJ, Barrot M, DiLeone RJ, Eisch AJ, Gold SJ, Monteggia LM (2002). Neurobiology of depression. Neuron.

[151] Nguyen EC, McCracken KA, Liu Y, Pouw B, Matsumoto RR (2005). Involvement of sigma (σ) receptors in the acute actions of methamphetamine: receptor binding and behavioral studies. Neuropharmacology.

[152] Nobile M, Lagostena L (1998). A discriminant block among K^+^ channel types by phenytoin in neuroblastoma cells. Br. J. Pharmacol.

[153] Noda Y, Kamei H, Kamei Y, Nagai T, Nishida M, Nabeshima T (2000). Neurosteroids ameliorate conditioned fear stress: an association with sigma_1_ receptors. Neuropsychopharmacology.

[154] Novakova M, Bruderova V, Sulova Z, Kopacek J, Lacinova L, Kvetnansky R, Vasku A, Kaplan P, Krizanova O, Jurkovicova D (2007). Modulation of expression of the sigma receptors in the heart of rat and mouse in normal and pathological conditions. Gen. Physiol. Biophys.

[155] Nudmamud-Thanoi S, Reynolds GP (2004). The NR1 subunit of the glutamate/NMDA receptor in the superior temporal cortex in schizophrenia and affective disorders. Neurosci. Lett.

[156] Nuwayhid SJ, Werling LL (2006). Sigma_2_ (σ_2_) receptors as a target for cocaine action in the rat striatum. Eur. J. Pharmacol.

[157] Okuyama S, Imagawa Y, Tomisawa K (1996). Behavioral Evidence for Modulation by Sigma Ligands of (+)MK-801-induced Hyperlocomotion in Monoamine-depleted Mice. Neuropharmacology.

[158] Okuyama S, Nakazato A (1996). NE-100: a novel sigma receptor antagonist. CNS Drug Rev.

[159] Pal A, Hajipour AR, Fontanilla D, Ramachandran S, Chu UB, Mavlyutov T, Ruoho AE (2007). Identification of regions of the σ-1 receptor ligand binding site using a novel photoprobe. Mol. Pharmacol.

[160] Palacios G, Muro A, Vela JM, Molina-Holgado E, Guitart X, Ovalle S, Zamanillo D (2003). Immunohistochemical localization of the σ_1_-receptor in oligodendrocytes in the rat central nervous system. Brain Res.

[161] Pan YX, Mei J, Xu J, Wan BL, Zuckerman A, Pasternak GW (1998). Cloning and characterization of a mouse σ_1_ receptor. J. Neurochem.

[162] Peeters M, Romieu P, Maurice T, Su TP, Maloteaux JM, Hermans E (2004). Involvement of the sigma_1_ receptor in the modulation of dopaminergic transmission by amantadine. Eur. J. Neurosci.

[163] Phan VL, Miyamoto Y, Nabeshima T, Maurice T (2005). Age-related expression of σ_1_ receptors and antidepressant efficacy of a selective agonist in the senescence-accelerated (SAM) mouse. J. Neurosci. Res.

[164] Poncelet M, Santucci V, Paul R, Gueudet C, Lavastre S, Guitard J, Steinberg R, Terranova JP, Breliere JC, Soubrie P (1993). Neuropharmacological profile of a novel and selective ligand of the sigma site: SR 31742A. Neuropharmacology.

[165] Quirion R, Bowen WD, Itzhak Y, Junien JL, Musacchio JM, Rothman RB, Su TP, Tam SW, Taylor DP (1992). A proposal for the classification of σ binding sites. Trends Pharmacol. Sci.

[166] Riedel G, Platt B, Micheau J (2003). Glutamate receptor function in learning and memory. Behav. Brain Res.

[167] Rogoz Z, Skuza G (2006). Mechanism of synergistic action following co-treatment with pramipexole and fluoxetine or sertraline in the forced swimming test in rats. Pharmacol. Rep.

[168] Romieu P, Martin-Fardon R, Maurice T (2000). Involvement of the sigma_1_ receptor in the cocaine-induced conditioned place preference. Neuroreport.

[169] Romieu P, Phan VL, Martin-Fardon R, Maurice T (2002). Involvement of the sigma_1_ receptor in cocaine-induced conditioned place preference: possible dependence on dopamine uptake blockade. Neuropsychopharmacology.

[170] Romieu P, Meunier J, Garcia D, Zozime N, Martin-Fardon R, Bowen WD, Maurice T (2004). The sigma_1_ (*σ_1_*) receptor activation is a key step for the reactivation of cocaine conditioned place preference by drug priming. Psychopharmacology (Berl.).

[171] Romieu P, Lucas M, Maurice T (2006). σ_1_ receptor ligands and related neuroactive steroids interfere with the cocaine-induced state of memory. Neuropsychopharmacology.

[172] Ronsisvalle G, Marrazzo A, Prezzavento O, Pasquinucci L, Falcucci B, Di Toro RD, Spampinato S (2000). Substituted 1-phenyl-2-cyclopropylmethylamines with high affinity and selectivity for sigma sites. Bioorg. Med. Chem.

[173] Ronsisvalle G, Marrazzo A, Prezzavento O, Cagnotto A, Mennini T, Parenti C, Scoto GM (2001). Opioid and sigma receptor studies. New developments in the design of selective sigma ligands. Pure Appl. Chem.

[174] Roth MD, Whittaker KM, Choi R, Tashkin DP, Baldwin GC (2005). Cocaine and σ-1 receptors modulate HIV infection, chemokine receptors, and the HPA axis in the huPBL-SCID model. J. Leukoc. Biol.

[175] Rothman RB, Baumann MH (2003). Monoamine transporters and psychostimulant drugs. Eur. J. Pharmacol.

[176] Rückert NG, Schmidt WJ (1993). The *σ* receptor ligand 1,3-di-(2-tolyl)guanidine in animal models of schizophrenia. Eur. J. Pharmacol.

[177] Rush AM, Elliott JR (1997). Phenytoin and carbamazepine: differential inhibition of sodium currents in small cells from adult rat dorsal root ganglia. Neurosci. Lett.

[178] Schwarz S, Pohl P, Zhou GZ (1989). Steroid binding at sigma-"opioid" receptors. Science.

[179] Seth P, Leibach FH, Ganapathy V (1997). Cloning and structural analysis of the cDNA and the gene encoding the murine type 1 sigma receptor. Biochem. Biophys. Res. Commun.

[180] Seth P, Fei YJ, Li HW, Huang W, Leibach FH, Ganapathy V (1998). Cloning and functional characterization of a *σ* receptor from rat brain. J. Neurochem.

[181] Seth P, Ganapathy ME, Conway SJ, Bridges CD, Smith SB, Casellas P, Ganapathy V (2001). Expression pattern of the type 1 sigma receptor in the brain and identity of critical anionic amino acid residues in the ligand-binding domain of the receptor. Biochim. Biophys. Acta.

[182] Shin EJ, Nah SY, Kim WK, Ko KH, Jhoo WK, Lim YK, Cha JY, Chen CF, Kim HC (2005). The dextromethorphan analog dimemorfan attenuates kainate-induced seizures *via* *σ*_1_ receptor activation: comparison with the effects of dextromethorphan. Br. J. Pharmacol.

[183] Silvers JM, Wallace DR, Harrod SB, Mactutus CF, Booze RM (2006). Prenatal cocaine alters dopamine and sigma receptor binding in nucleus accumbens and striatum in dams and adolescent offspring. Neurotoxicol. Teratol.

[184] Skuza G (1999). Effect of sigma ligands on the cocaine-induced convulsions in mice. Pol. J. Pharmacol.

[185] Skuza G, Rogoz Z (2002). A potential antidepressant activity of SA4503, a selective *σ*_1_ receptor agonist. Behav. Pharmacol.

[186] Skuza G, Rogoz Z (2006). The synergistic effect of selective sigma receptor agonists and uncompetitive NMDA receptor antagonists in the forced swim test in rats. J. Physiol. Pharmacol.

[187] Skuza G, Rogoz Z (2006). Effect of BD 1047, a sigma_1_ receptor antagonist, in the animal models predictive of antipsychotic activity. Pharmacol. Rep.

[188] Soriani O, Foll FL, Roman F, Monnet FP, Vaudry H, Cazin L (1999). A-Current down-modulated by *σ* receptor in frog pituitary melanotrope cells through a G protein-dependent pathway. J. Pharmacol. Exp. Ther.

[189] Soriani O, Le Foll F, Galas L, Roman F, Vaudry H, Cazin L (1999). The σ-ligand (+)-pentazocine depresses M current and enhances calcium conductances in frog melanotrophs. Am. J. Physiol.

[190] South SM, Kohno T, Kaspar BK, Hegarty D, Vissel B, Drake CT, Ohata M, Jenab S, Sailer AW, Malkmus S, Masuyama T, Horner P, Bogulavsky J, Gage FH, Yaksh TL, Woolf CJ, Heinemann SF, Inturrisi CE (2003). A conditional deletion of the NR1 subunit of the NMDA receptor in adult spinal cord dorsal horn reduces NMDA currents and injury-induced pain. J. Neurosci.

[191] Stefanski R, Justinova Z, Hayashi T, Takebayashi M, Goldberg SR, Su TP (2004). Sigma_1_ receptor upregulation after chronic methamphetamine self-administration in rats: a study with yoked controls. Psychopharmacology (Berl.).

[192] Stone JM, Arstad E, Erlandsson K, Waterhouse RN, Ell PJ, Pilowsky LS (2006). [^123^I]TPCNE-a novel SPET tracer for the sigma-1 receptor: first human studies and *in vivo* haloperidol challenge. Synapse.

[193] Su TP, London ED, Jaffe JH (1988). Steroid binding at σ receptors suggests a link between endocrine, nervous, and immune systems. Science.

[194] Su TP, Hayashi T (2001). Cocaine affects the dynamics of cytoskeletal proteins *via* sigma_1_ receptors. Trends Pharmacol. Sci.

[195] Takahashi S, Sonehara K, Takagi K, Miwa T, Horikomi K, Mita N, Nagase H, Iizuka K, Sakai K (1999). Pharmacological profile of MS-377, a novel antipsychotic agent with selective affinity for σ receptors. Psychopharmacology (Berl).

[196] Takahashi S, Miwa T, Horikomi K (2000). Involvement of σ^1^ receptors in methamphetamine-induced behavioral sensitization in rats. Neurosci. Lett.

[197] Takahashi S, Horikomi K, Kato T (2001). MS-377, a novel selective σ_1_ receptor ligand, reverses phencyclidine-induced release of dopamine and serotonin in rat brain. Eur. J. Pharmacol.

[198] Takebayashi M, Hayashi T, Su TP (2002). Nerve growth factor-induced neurite sprouting in PC12 cells involves *σ*-1 receptors implications for antidepressants. J. Pharmacol. Exp. Ther.

[199] Takebayashi M, Hayashi T, Su TP (2004). σ-1 receptors potentiate epidermal growth factor signaling towards neuritogenesis in PC12 cells: potential relation to lipid raft reconstitution. Synapse.

[200] Tam SW, Steinfels GF, Gilligan PJ, Schmidt WK, Cook L (1992). DuP 734 [1-(cyclopropylmethyl)-4-(2'(4''-fluorophenyl)-2'-oxoethyl)- piperidine HBr] a *sigma* and 5-hydroxytryptamine_2_ receptor antagonist: receptor-binding, electrophysiological and neuropharmacological profiles. J. Pharmacol. Exp. Ther.

[201] Taylor DP, Eison MS, Moon SL, Schlemmer RF Jr, Shukla UA, VanderMaelen CP, Yocca FD, Gallant DJ, Behling SH, Boissard CG (1993). A role for σ binding in the antipsychotic profile of BMY 14802?. NIDA. Res. Monogr.

[202] Todorovic SM, Lingle CJ (1998). Pharmacological properties of T-type Ca^2+^ current in adult rat sensory neurons: effects of anticonvulsant and anesthetic agents. J. Neurophysiol.

[203] Tottori K, Miwa T, Uwahodo Y, Yamada S, Nakai M, Oshiro Y, Kikuchi T, Altar CA (2001). Antidepressant-like responses to the combined sigma and 5-HT_1A_ receptor agonist OPC-14523. Neuropharmacology.

[204] Ueda H, Inoue M, Yoshida A, Mizuno K, Yamamoto H, Maruo J, Matsuno K, Mita S (2001). Metabotropic neurosteroid/*σ*-receptor involved in stimulation of nociceptor endings of mice. J. Pharmacol. Exp. Ther.

[205] Ujike H, Kanzaki A, Okumura K, Akiyama K, Otsuki S (1992). Sigma (σ) antagonist BMY 14802 prevents methamphetamine-induced sensitization. Life Sci.

[206] Ujike H, Kuroda S, Otsuki S (1996). *σ* Receptor antagonists block the development of sensitization to cocaine. Eur. J. Pharmacol.

[207] Ukai M, Maeda H, Nanya Y, Kameyama T, Matsuno K (1998). Beneficial effects of acute and repeated administrations of σ receptor agonists on behavioral despair in mice exposed to tail suspension. Pharmacol. Biochem. Behav.

[208] Urani A, Roman FJ, Phan VL, Su TP, Maurice T (2001). The antidepressant-like effect induced by *σ*_1_-receptor agonists and neuroactive steroids in mice submitted to the forced swimming test. J. Pharmacol. Exp. Ther.

[209] Urani A, Romieu P, Roman FJ, Maurice T (2002). Enhanced antidepressant effect of sigma_1_ (σ_1_) receptor agonists in β_25-35_--amyloid peptide-treated mice. Behav. Brain Res.

[210] Urani A, Romieu P, Portales-Casamar E, Roman FJ, Maurice T (2002). The antidepressant-like effect induced by the sigma_1_ (*σ*_1_) receptor agonist igmesine involves modulation of intracellular calcium mobilization. Psychopharmacology (Berl.).

[211] Urani A, Romieu P, Roman FJ, Yamada K, Noda Y, Kamei H, Manh TH, Nagai T, Nabeshima T, Maurice T (2004). Enhanced antidepressant efficacy of σ_1_ receptor agonists in rats after chronic intracerebroventricular infusion of β-amyloid-(1-40) protein. Eur. J. Pharmacol.

[212] van Berckel BNM (2003). glutamate and schizophrenia. Curr. Neuropharmacol.

[213] Volz HP, Stoll KD (2004). Clinical trials with sigma ligands. Pharmacopsychiatry.

[214] Walker JM, Bowen WD, Walker FO, Matsumoto RR, De Costa B, Rice KC (1990). Sigma receptors: biology and function. Pharmacol. Rev.

[215] Walker JM, Bowen WD, Patrick SL, Williams WE, Mascarella SW, Bai X, Carroll FI (1993). A comparison of (-)-deoxybenzomorphans devoid of opiate activity with their dextrorotatory phenolic counterparts suggests role of *σ*_2_ receptors in motor function. Eur. J. Pharmacol.

[216] Wang D, Noda Y, Tsunekawa H, Zhou Y, Miyazaki M, Senzaki K, Nitta A, Nabeshima T (2007). Role of N-methyl-D-aspartate Receptors in Antidepressant-Like Effects of *σ*_1_ Receptor Agonist 1-(3,4-dimethoxyphenethyl)-4-(3-phenylpropyl)piperazine Dihydrochloride (SA-4503) in Olfactory Bulbectomized Rats. J. Pharmacol. Exp. Ther.

[217] Wang HH, Chien JW, Chou YC, Liao JF, Chen CF (2003). Anti-amnesic effect of dimemorfan in mice. Br. J. Pharmacol.

[218] Wang J, Mack AL, Coop A, Matsumoto RR (2007). Novel sigma (σ) receptor agonists produce antidepressant-like effects in mice. Eur. Neuropsychopharmacol.

[219] Waterhouse RN, Chang RC, Atuehene N, Collier TL (2007). *In vitro* and *in vivo* binding of neuroactive steroids to the sigma-1 receptor as measured with the positron emission tomography radioligand [^18^F]FPS. Synapse.

[220] Wilke RA, Lupardus PJ, Grandy DK, Rubinstein M, Low MJ, Jackson MB (1999). K^+^ channel modulation in rodent neurohypophysial nerve terminals by sigma receptors and not by dopamine receptors. J. Physiol.

[221] Witkin JM, Terry P, Menkel M, Hickey P, Pontecorvo M, Ferkany J, Katz JL (1993). Effects of the selective *sigma* receptor ligand, 6-[6-(4-hydroxypiperidinyl)hexyloxy]-3-methylflavone (NPC 16377), on behavioral and toxic effects of cocaine. J. Pharmacol. Exp. Ther.

[222] Yagasaki Y, Numakawa T, Kumamaru E, Hayashi T, Su TP, Kunugi H (2006). Chronic antidepressants potentiate *via* sigma-1 receptors the brain-derived neurotrophic factor-induced signaling for glutamate release. J. Biol. Chem.

[223] Zhang D, Zhang L, Tang Y, Zhang Q, Lou D, Sharp FR, Zhang J, Xu M (2005). Repeated cocaine administration induces gene expression changes through the dopamine D_1_ receptors. Neuropsychopharmacology.

[224] Zhang H, Cuevas J (2005). σ Receptor activation blocks potassium channels and depresses neuroexcitability in rat intracardiac neurons. J. Pharmacol. Exp. Ther.

